# Methods to estimate marine functional connectivity: A primer

**DOI:** 10.1002/eap.70273

**Published:** 2026-06-24

**Authors:** Anna M. Sturrock, Susanne E. Tanner, Sophie Arnaud‐Haond, Jacopo Aguzzi, Francisco R. Barboza, Maria Beger, Andreu Blanco, Deirdre Brophy, Marta Carreton, Amber‐Robyn Childs, Federica Costantini, Oscar E. Gaggiotti, Bronwyn M. Gillanders, José M. González‐Irusta, Katell Guizien, Tamar Guy‐Haim, Stefanie Haase, Ewan Hunter, Jonne Kotta, Geneviève Lacroix, Rafet Ç. Öztürk, Angel Pérez‐Ruzafa, Patrick Reis‐Santos, Cynthia Riginos, Gil Rilov, Buki Rinkevich, Guiomar Rotllant, David H. Secor, Szymon Smoliński, Clive N. Trueman, Benjamin D. Walther, Audrey M. Darnaude

**Affiliations:** ^1^ School of Life Sciences, University of Essex Essex UK; ^2^ Marine and Environmental Sciences Centre, Department of Biology, Faculty of Sciences University of Lisbon Lisbon Portugal; ^3^ Ifremer, Station de Sète Sète Cedex France; ^4^ Institut de Ciències del Mar (ICM‐CSIC) Barcelona Catalan Country Spain; ^5^ Estonian Marine Institute, University of Tartu Tallinn Estonia; ^6^ School of Biology, Faculty of Biological Sciences University of Leeds Leeds UK; ^7^ School of the Environment, University of Queensland Brisbane Queensland Australia; ^8^ Centro de Investigación Mariña, Future Oceans Lab Universidade de Vigo Vigo Spain; ^9^ Marine and Freshwater Research Centre, Atlantic Technological University (ATU), ATU Galway City Galway Ireland; ^10^ ICATMAR Barcelona Spain; ^11^ Department of Ichthyology and Fisheries Science Rhodes University Makhanda Eastern Cape South Africa; ^12^ Department of Biological, Geological and Environmental Science University of Bologna Ravenna Italy; ^13^ Centre for Biological Diversity School of Biology, University of St Andrews St Andrews UK; ^14^ School of Biological Sciences, Adelaide University Adelaide South Australia Australia; ^15^ Instituto Español de Oceanografía (CSIC), Centro Oceanografico de Santander Santander Spain; ^16^ CNRS‐Sorbonne Université, Laboratoire d'Ecogéochimie des Environnements Benthiques LECOB Banyuls‐sur‐Mer France; ^17^ National Institute of Oceanography, Israel Oceanographic and Limnological Research Haifa Israel; ^18^ Thünen Institute of Baltic Sea Fisheries Rostock Germany; ^19^ Fisheries and Aquatic Ecosystems Branch, Agri‐Food and Biosciences Institute Belfast UK; ^20^ Royal Belgium Institute of Natural Sciences Brussels Belgium; ^21^ Department of Fisheries Technology Engineering Karadeniz Technical University Trabzon Turkey; ^22^ Department of Ecology and Hydrology and Regional Campus of International Excellence “Mare Nostrum” University of Murcia Murcia Spain; ^23^ Australian Institute of Marine Science Cape Cleveland Queensland Australia; ^24^ University of Maryland Center for Environmental Science (UMCES) Cambridge Maryland USA; ^25^ Department of Fisheries Resources National Marine Fisheries Research Institute Gdynia Poland; ^26^ Ocean and Earth Science University of Southampton Southampton UK; ^27^ Department of Life Sciences Texas A&M University – Corpus Christi Corpus Christi Texas USA; ^28^ MARBEC, University of Montpellier, CNRS, IRD, Ifremer Montpellier France

**Keywords:** bioinvasions, dispersal, ecological modeling, fisheries management, genetics, marine protected areas, migration, movement, otolith chemistry, tagging

## Abstract

Organism movement is a key process in the transfer of individuals, genes, functional traits, matter, and energy among habitat patches, at sea and across the land–sea interface. The resulting fluxes, collectively termed marine functional connectivity (MFC), underpin planetary health and an array of ecosystem services. The ecological and economic impacts of rapid environmental change, including climate change, overexploitation, habitat loss and fragmentation, and the global transport of nonindigenous species make accurate estimation and prediction of MFC patterns paramount. However, estimating MFC is challenging given the relative inaccessibility of the oceans and the small size of many of the organisms and life stages with the highest dispersal potential. Here, we provide a methodological roadmap to help researchers and stakeholders understand, use, and integrate different tools to estimate organism movement and connectivity, focusing on (1) tagging and telemetry, (2) analysis of chemical markers in body tissues and structures, (3) genetics, and (4) numerical modeling. We describe method strengths and weaknesses, and the spatiotemporal resolution and scale of resulting connectivity estimates. Ancillary and emerging methods to estimate MFC are also reviewed. We then present case studies that have successfully applied or integrated different methods, particularly to support (1) marine protected area design, (2) global change predictions, focusing on climate change and bioinvasions, and (3) fisheries management. Finally, we highlight methodological innovations and concepts that promise to transform MFC research in the future.

## INTRODUCTION

Marine functional connectivity (MFC) refers to the transfer of individuals, genes, functional traits, matter, and energy resulting from the movements of organisms at sea and across the land–sea interface (Darnaude et al., 2026). MFC operates across all spatial and temporal scales, with passive dispersal and active migration of organisms linking areas at daily, seasonal, demographic, and evolutionary timescales (Rountree et al., 2020). Collectively, these movements underpin the oceans' ecosystem functions and services, including fisheries, carbon sequestration, and nutrient cycling (Preston et al., 2025), estimated at almost a million USD per hectare per year (De Groot et al., 2012).

In the context of rapid global change, it is paramount to develop an integrated methodological “toolkit” that can effectively measure and predict MFC, and to incorporate this information into marine management and conservation. Climate change is driving large shifts in species distribution, phenology, and MFC patterns, particularly through changing water temperature and ocean circulation patterns (Fromentin et al., 2014; Marshall & Alvarez‐Noriega, 2020). Other anthropogenic activities can also artificially augment MFC, with increasing global trade and transport accelerating the spread of nonindigenous species across the globe (Bailey, 2015; Seebens et al., 2013). Importantly, environmental change—and the combined effects of multiple stressors—can be nonlinear and difficult to predict (Lacroix et al., 2018), making it challenging to design management actions that meet both current and future needs (Bindoff et al., 2019; Crowder et al., 2000; McLeod et al., 2009). However, as discussed in *Applications: Now and in the future*, spatial planners and resource managers are increasingly leveraging connectivity data to try to buffer some of this unpredictability, particularly in marine protected area (MPA) design (Gardner et al., 2024) and fisheries stock assessment (Cadrin et al., 2023).

Estimating and monitoring MFC is inherently challenging, because marine environments are vast, open, three‐dimensional spaces that are difficult and expensive to access. Real‐time monitoring through remote and in situ observation can help to quantify changes in key biotic and abiotic parameters, but the tools are often technologically immature or unaffordable for widespread implementation (Aguzzi et al., 2024; Bellou et al., 2021). Furthermore, the dispersive stages of marine species are often tiny and exhibit high mortality rates, making them difficult to sample and track, and connectivity‐relevant traits (e.g., pelagic larval duration [PLD], intergenerational time) vary considerably among taxa, making it difficult to make generalized predictions across species and ecosystems (Bradbury et al., 2009; Cowen & Sponaugle, 2009). MFC also explicitly considers the influence of organism morphology, behavior (e.g., swimming capacity), and other biological traits (e.g., reproduction mode) on connectivity patterns. While challenging to quantify, these traits can significantly influence flux direction and strength, all the way from dispersing larvae to actively migrating megafauna (Darnaude et al., 2024). MFC is also distinct from genetic, population, and demographic connectivity (Cowen & Sponaugle, 2009; Secor, 2015), by not only considering gene flow, but also more transient exchanges that can result in large fluxes of biomass and energy (e.g., the “whale pump,” Roman & McCarthy, 2010).

**FIGURE 1:**
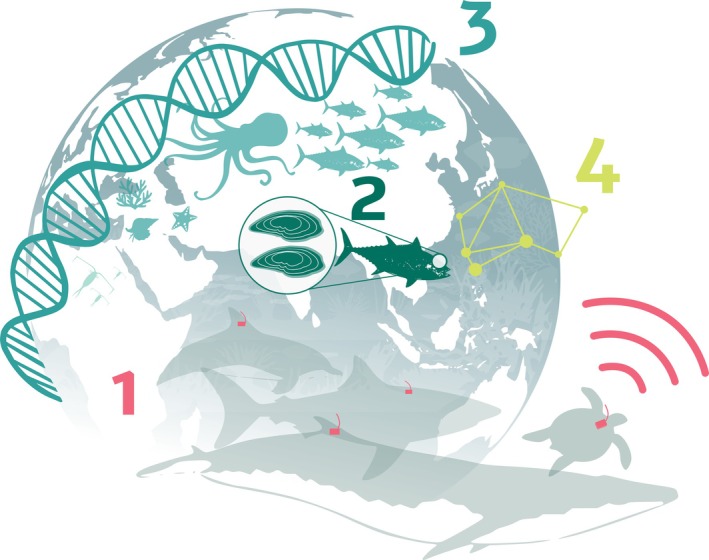
Conceptual depiction of the main methods reviewed in this paper: (1) Tagging and telemetry, (2) chemical markers, (3) genetics, (4) numerical modeling. Artwork by Siegrid Mons (Siegrid Design), Scientific Graphic Designer.

Here, we review the main methodologies used to estimate or predict MFC: (1) tagging and telemetry, (2) analysis of chemical markers in body tissues and structures, (3) genetics, and (4) numerical modeling (Figure 1). Our aim is to provide a methodological roadmap to help researchers and stakeholders understand, use, and integrate different tools to estimate MFC patterns and to build capacity and knowledge across often siloed research fields. Each method has a suite of underlying assumptions, strengths, and weaknesses that determine the scale, accuracy, and precision of MFC estimation (summarized in Table 1). We then explore how these methods can be integrated and applied to support (1) MPA design, (2) global change predictions, with a particular focus on climate change and bioinvasions, and (3) fisheries management. Finally, we highlight the methodological innovations that will transform MFC science in the future.

**TABLE 1:** Strengths and weaknesses associated with the various methods used to estimate marine functional connectivity, with the main ones highlighted in bold.

Method	Spatial resolution	Temporal resolution	Ease of use	Additional information
**Tagging and telemetry**				
Strengths	Very high resolution (electronic tags). Global coverage can be enabled via satellite technology. **Can directly track individual movements with high certainty**.	Very high temporal resolution (electronic tags), often capable of logging data at <1‐min resolution. Flexible programming can allow temporally targeted recording.	Tag cost, size and mass is decreasing, increasing broad applicability. Increasing innovation in data recovery, coverage and sharing via global arrays and networks (e.g., OTN).	Sensors can link movements to internal and external covariates (e.g., temperature, heart rate, predation). Excellent for public engagement (e.g., websites showing the tracks of an individually named shark).
Weaknesses	Acoustic tags: limited by receiver detection capability and array extent. Mark–recapture: limited to two‐point locations and can be biased by sampling or fishing effort. DSTs: Interpretation dependent on availability, scale, and accuracy of reference environmental datasets.	Electronic tags limited by battery life and memory capacities.	Animal needs to be handled (welfare, training, regulation considerations). **Many methods require tag recovery**. Electronic tags and setting up/maintaining receiver arrays is costly. Marine pollution (e.g., lithium batteries).	Generally low sample sizes due high field and tag costs. Generally limited to larger bodied animals with hard body parts to attach the tag to. Some satellite tags rely on animal surfacing to transmit data, excluding many species.
**Chemical markers**				
Strengths	Spatially explicit information can be extracted, but the resolution depends on marker and system. Typically the highest resolution information obtained across the land–sea interface using Sr/Ca, Ba/Ca, ^87^Sr/^86^Sr, δ^18^O.	**Archival tissues allow lifetime (and sometimes transgenerational) movements to be reconstructed, including early life stages too small to tag externally**.	Many tissues are inert so markers are permanently stored (easy preservation). Some markers (e.g., δ^13^C and δ^15^N in soft tissues) are relatively low cost and routine to analyze.	Often archival tissues can also provide additional insights into individual health (e.g., growth rate, age, health, reproduction, metabolic rate).
Weaknesses	**Relationship between environment and tissue is often noisy or unknown, or there are limited chemical differences among areas, reducing spatial resolution**. Difficult/expensive to obtain reference samples with sufficient coverage to generate appropriate isoscapes or chemoscapes (predictive maps).	Temporal instability in many markers can require cohort‐matched reference libraries increasing time and cost. In adult life stages, slow tissue growth often reduces the temporal resolution possible for probe‐based analyses.	Many tissues take considerable time and training to prepare for analysis. Many analyses require expert technicians to run the instrument, increasing cost and wait times.	Often tissues obtained via lethal sampling (ethical considerations).
**Genetics**				
Strengths	High confidence to detect absence of connectivity. Different methods can be applied across spatial scales from highly local (e.g., within MPA) to global.	Different temporal scales can be resolved using different methods (Figure 2).	Protocols for developing SNP‐based nuclear markers or microsatellites are now well established and implemented by most molecular labs.	Can give information on population or individual origin. **Can be used for all species and life stages, and only a small quantity of material is needed to obtain sufficient DNA (i.e., nonlethal)**
Weaknesses	**Even limited gene flow can erode genetic differences so that assignment tests and demographic inference methods cannot reliably distinguish connectivity levels greater than ~1 migrant per generation**. Parentage analysis requires exhaustive sampling and thus is only feasible for species with restricted spatial distributions and moderate to small population sizes.	With the exception of parentage analyses, timescales are usually too coarse to detect short‐term changes in connectivity, that are often the ones most relevant to management and conservation. To detect short‐term changes in connectivity using parentage methods, sampling needs to be carried out over multiple time points.	For some species and preservation methods there can be difficulties in extracting sufficient quantities of high enough quality of DNA. Depending on the markers and molecular protocols used, analyses can be expensive.	Genetic homogeneity does not necessarily imply connectivity sufficient to affect census population size (i.e., high evolutionary connectivity levels may not be ecologically relevant).
**Numerical modeling**				
Strengths	Biophysical dispersal model outputs combined with matrix projection can be used to provide information on connectivity structure, including directionality of flows.	Biophysical models are often created at high temporal resolution. **Modeling allows both backtracking and forecasting (e.g., predicting effect of climate change and alternate management actions)**.	Models are increasingly available in multiple open source coding languages (e.g., Python, R). No requirement to sample any organisms (cheaper and more ethically sound).	SDMs can inform biophysical models by providing the predicted suitable habitat of the focus species. Predictive maps can be an effective tool for science communication and outreach, but caveats and uncertainties need to be well communicated.
Weaknesses	Hydrodynamical models may not match the spatial extent or resolution of the connectivity pattern being predicted. Lack of three‐dimensional data predictors at adequate spatial resolution can limit model predictions.	Hydrodynamical forcings may not match the temporal window or resolution of the connectivity pattern being predicted.	Requires coding skills and often high‐performance computer clusters (expensive, and time/memory intensive). Requires underlying hydrodynamical models, which are not always available at appropriate spatial and temporal resolution. Requires biological data (reproduction, larval duration, growth rate) from all species to be included in the models, which are often lacking.	**It is difficult to calibrate model without accurate estimates of biological traits (e.g., spawning period/timing, PLD, behavior) which are often lacking or inaccurate**. Lack of proper validation (e.g., trajectories) and error estimation.

*Note*: For details on specific techniques, refer to the main text and Sturrock and Tanner (2026b).

Abbreviations: DST, data storage tag; MPA, marine protected area; OTN, Ocean Tracking Network; PLD, pelagic larval duration; SDM, species distribution model; SNP, single‐nucleotide polymorphism.

## AN OVERVIEW OF CONNECTIVITY ESTIMATION METHODS

**FIGURE 2:**
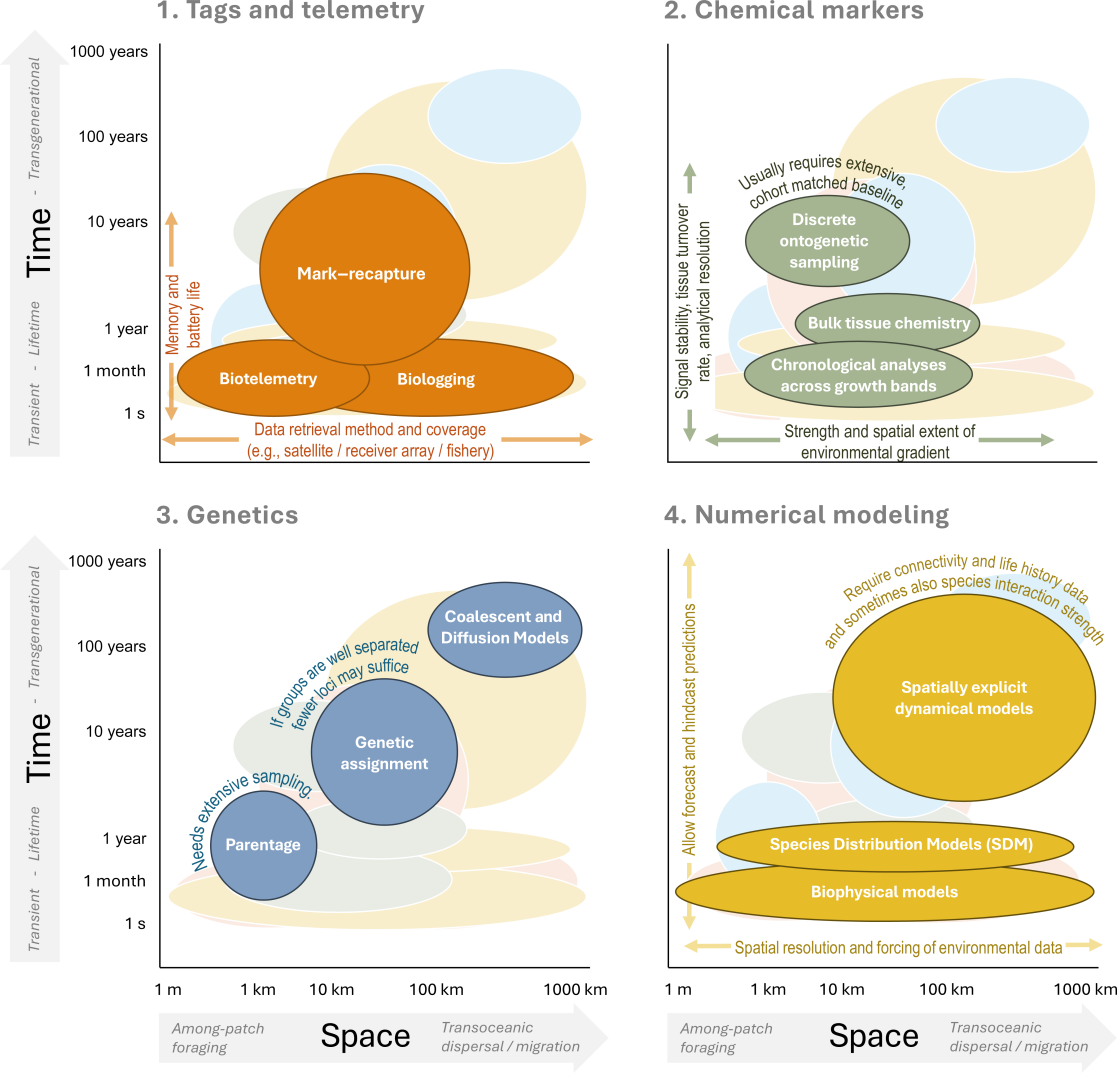
Overview of the spatiotemporal resolution of the connectivity estimates obtained from the methods reviewed in this paper, where the spatial extent of the flux in question is—in turn—determined by the distance between patches, inspired by Jones et al. (2009) and Tanner et al. (2016). Method‐specific considerations are highlighted, with a particular focus on those influencing the resolution or extent of resulting marine functional connectivity (MFC) estimates.

Here, we review the main methods used to assess MFC, their key advantages and disadvantages (Table 1), and differences in their spatiotemporal resolution (Figure 2). Sturrock and Tanner (2026b) and Appendix S1: Section S1 provide additional details to assist new and existing users in tool selection, study design, and data interpretation, and also give an overview of ancillary methods that support MFC estimation, such as environmental DNA (eDNA) and population markers such as morphometrics. Note that in this review we use the term “migration” in its most flexible way, including transient and periodic migrations as well as “one way” migrations (immigration or emigration) that result in gene flow (Whitlock & McCauley, 1999).

### Tagging and telemetry

#### Methods

In this section, we refer to animal‐attached tags to track or reconstruct their movements. The earliest tagging work used mark–recapture methods to identify movements between marking and recapture sites, an approach that remains popular today (Secor, 2015). Mark–recapture studies typically use applied external marks (e.g., fin clips, branding, sequentially numbered tags), internal marks (described in *Chemical markers*), or non‐transmitting tags such as coded wire tags (Hazen et al., 2012). Today, sophisticated electronic tags allow animal movements to be tracked or reconstructed at high spatiotemporal resolution using biologging and biotelemetry.

Biologging involves tagging an individual with an electronic device containing sensors that systematically log data in its memory at particular timestamps (Cooke et al., 2012; Thorstad et al., 2014; Watanabe & Papastamatiou, 2023). These archival or data storage tags (DSTs) typically measure pressure (a proxy for depth), temperature and sometimes—with appropriate calibration (Brownscombe et al., 2019)—also salinity and dissolved oxygen (Coffey & Holland, 2015). For many DSTs, direct positions are not available and individual tracks are reconstructed from environmental records downloaded after the animal or tag is recovered (reviewed in Gatti et al., 2021). To improve tag recovery rates, DSTs are increasingly housed in a positively buoyant flotation jacket that floats to the surface where they are more likely to be recovered, for example, when washed onto beaches (De Pontual et al., 2023). The active release of DSTs, programmed to “pop‐off” after a predetermined time, can further boost data retrieval (e.g., Nielsen et al., 2023). Satellite tags, which have a hybrid design of biologging and biotelemetry (Cooke et al., 2021; Matley et al., 2022), store data in their memory and transmit/relay data on the animal's position and its environmental experience (via multi‐sensor capabilities) to satellite receivers either when the animal surfaces or when the tag is released to the surface (e.g., via pop‐off mechanisms). Biologging electronic devices can also be coupled with cameras directly attached to the animal to understand how cues within an individual's ecofield (the portion of habitat in their direct sensorial range; Farina & Belgrano, 2004) influences their movement and behavior (Hays, 2015; Moll et al., 2007; Wilmers et al., 2015).

Biotelemetry relies on tags that transmit a unique identity code (and potentially also auxiliary data such as temperature, pressure/depth, and acceleration in the case of sensor tags) to a remotely placed receiver (Heupel et al., 2006; Hussey et al., 2015; Vigo et al., 2021). For high‐resolution tracking across the land–sea interface and in the nearshore environment, biotelemetry using acoustic tags is a particularly popular approach (Hussey et al., 2015; Matley et al., 2022). Passive Integrated Transponder (PIT) tags are also frequently used to track animal movements through shallow estuarine environments (Colombano et al., 2020) and sometimes also at broader spatial scales (Ono et al., 2022). Biotelemetry allows the horizontal and vertical (via pressure/depth sensor tags) movements of tagged animals to be characterized based on detections across a network of moored receiver arrays (e.g., Rotllant et al., 2015; Vigo et al., 2021). Significant costs are associated with deployment and maintenance of these arrays, with fine‐scale coverage required in more complex habitats. Data recovery and sharing via biotelemetry are gradually improving through global tracking networks (Ellis et al., 2019; Matley et al., 2022) (Table 1). Other advances in biotelemetry include active tracking of tagged animals using robotic platforms (e.g., autonomous underwater vehicles, remotely operated vehicles, wave gliders) equipped with receivers (e.g., Lin et al., 2017; Masmitja et al., 2020) and “predator tags” that change the transmitted signal following digestion to avoid accidental tracking of the predator's movements (Klinard et al., 2019; Waters et al., 2025). Acoustic tags are also increasingly deployed in tandem with biologging tags or designed with integrated sensors that log environmental or physiological parameters experienced by the individual when outside the telemetry array. Animal recapture is necessary to extract and access these additional data records, but future tags could be designed to efficiently communicate with permanent infrastructural networks such as cabled observatories, neutrino telescopes, and decommissioned or to‐be‐decommissioned offshore structures such as oil and gas platforms (Aguzzi et al., 2019). Combining video (e.g., multiparametric video‐observatories that include environmental sensors; Aguzzi et al., 2020; Matabos et al., 2011) with acoustic tracking methods has the potential to provide information on fine‐scale movement and behavior, in particular for groups of organisms that are difficult to distinguish via image‐based approaches alone (e.g., shoaling fish) (Matley et al., 2023).

For both biologging and biotelemetry, tags can also include sensors to measure individual activity levels and movement across all three dimensions (e.g., using tri‐axial accelerometers for acceleration in three perpendicular axes; gyroscopes for angular velocity and magnetometers for orientation relative to the Earth's magnetic field; Andrzejaczek et al., 2019). When combined with heart rate, blood flow, and body temperature sensors (Williams & Ponganis, 2021), different behavioral and physiological states can be identified. By equipping individual animals with internal and external electronic tags boasting multiple sensors and biologging and telemetry capabilities, rich datasets can be generated to understand the biological and environmental drivers of animal movement (Brownscombe et al., 2019; Matley et al., 2022; Williams et al., 2020).

One disadvantage of applied tags is that they are rarely appropriate for small‐ (e.g., larvae) or soft‐bodied (e.g., salps, but see Fossette et al., 2016) organisms, and/or if the species' biology results in frequent tag loss, for example through molting (e.g., Curtis & McGaw, 2012). To circumvent some of these issues, internal tags tend to be used more on smaller bodied individuals, and tagging periods can be extended in decapods by targeting older individuals that exhibit extended intermoult periods (Hunter et al., 2013). For all tagging applications, body burden must be a central consideration, both for animal welfare reasons and to ensure natural behavior patterns. Technologies are becoming increasingly miniaturized, but tags able to record over multiple years (Brownscombe et al., 2019) and animal‐borne cameras (Deng et al., 2021) are still typically restricted to larger individuals.

#### Data analysis approaches

Traditional mark–recapture studies often release large numbers (hundreds to thousands) of individuals tagged with small, simple, cheap external tags. Smaller tags exert a lower burden on the subject animal, but there is a general expectation of low recapture rates (Secor, 2015). Mark–recapture studies can be used to estimate stock size, natural and fishing mortality (reviewed in Pine et al., 2003; Skalski et al., 2009), distribution patterns, home ranges, and seasonal habitat utilization (e.g., Robichaud & Rose, 2004), and to validate age and growth estimates (e.g., Beamish & McFarlane, 1983; Campana, 2005).

Electronic tagging studies are often restricted to smaller sample sizes (tens to hundreds), making broad generalizations difficult; however, tagging networks are rapidly changing this (Abecasis et al., 2018). Electronic tags often reveal unique behaviors and within‐population phenotypic diversity (Ward et al., 2016), and—when deployed at scale (hundred to thousands)—archival (De Pontual et al., 2023; Hunter et al., 2004) and acoustic (Itakura et al., 2021; Lilly et al., 2024) tags can identify population‐level behavioral “signatures” and patterns in habitat use.

Electronic tagging generates large volumes of complex and often highly autocorrelated data that are challenging to process, analyze and interpret (Joo et al., 2020). Fortunately, the expansion of biologging and biotelemetry in the last decade has coincided with rapid growth in the availability of statistical and programming techniques appropriate for addressing these challenges. The time series raw data can be analyzed using statistical approaches such as periodograms and waveforms (e.g., to assess movement frequency and timing), and the spatial extent of movements visualized using heat maps (Aguzzi et al., 2015). Data analyses often focus on seasonal, lunar, daily, and tidal behavioral patterns (e.g., Armannsson & Jónsson, 2012; Hunter et al., 2009; Subbey et al., 2008), and increasingly also their environmental niche (Raby et al., 2020; Righton et al., 2010). Network analyses have been instrumental in understanding connectivity patterns, although autocorrelation within spatial networks can pose challenges to understanding the patterns and drivers of space use (Jacoby et al., 2012; Jacoby & Freeman, 2016; Lédée et al., 2021). Mixed effects models (both generalized additive and linear forms) are also extremely popular, as they allow spatiotemporally autocorrelated variation, both within and between individuals, to be partitioned through the use of random effects (Whoriskey et al., 2019). Hidden Markov Models (HMMs) have been used extensively to estimate movement tracks from tagging records (Whoriskey et al., 2017) and can now also be fitted with individual‐level random effects (“mixed HMMs”) (McClintock, 2021). Some of the more popular R packages and functions to process, visualize, and analyze movement data include *Animal Tracking Toolbox*, *VTrack*, *momentuHMM*, *Actel*, *remora*, *HMMoce*, and *Flapper* (reviewed in Joo et al., 2020).

#### Temporal and spatial scales

Biologging and biotelemetry techniques cover a wide range of temporal and spatial scales. Depending on tag type and its battery life, memory capacity, and programmed logging regime, the recording duration can vary between days and years (Figure 2). Flexible programming of tags, particularly in the case of DSTs, allow for increased recording intensity during periods when behaviors of interest are expected, optimizing battery life and memory capacity. In terms of the temporal resolution achievable with tagging methods (i.e., the frequency of connectivity measurements through time), electronic tags operate at the highest resolution, with the potential to collect movement data at sub‐minute resolution. When environmental and physiological sensors are combined, researchers have the potential to identify individual responses to internal and external triggers, signposting the mechanisms shaping MFC at its finest scales (Goldbogen et al., 2019; Rudd et al., 2025).

**FIGURE 3:**
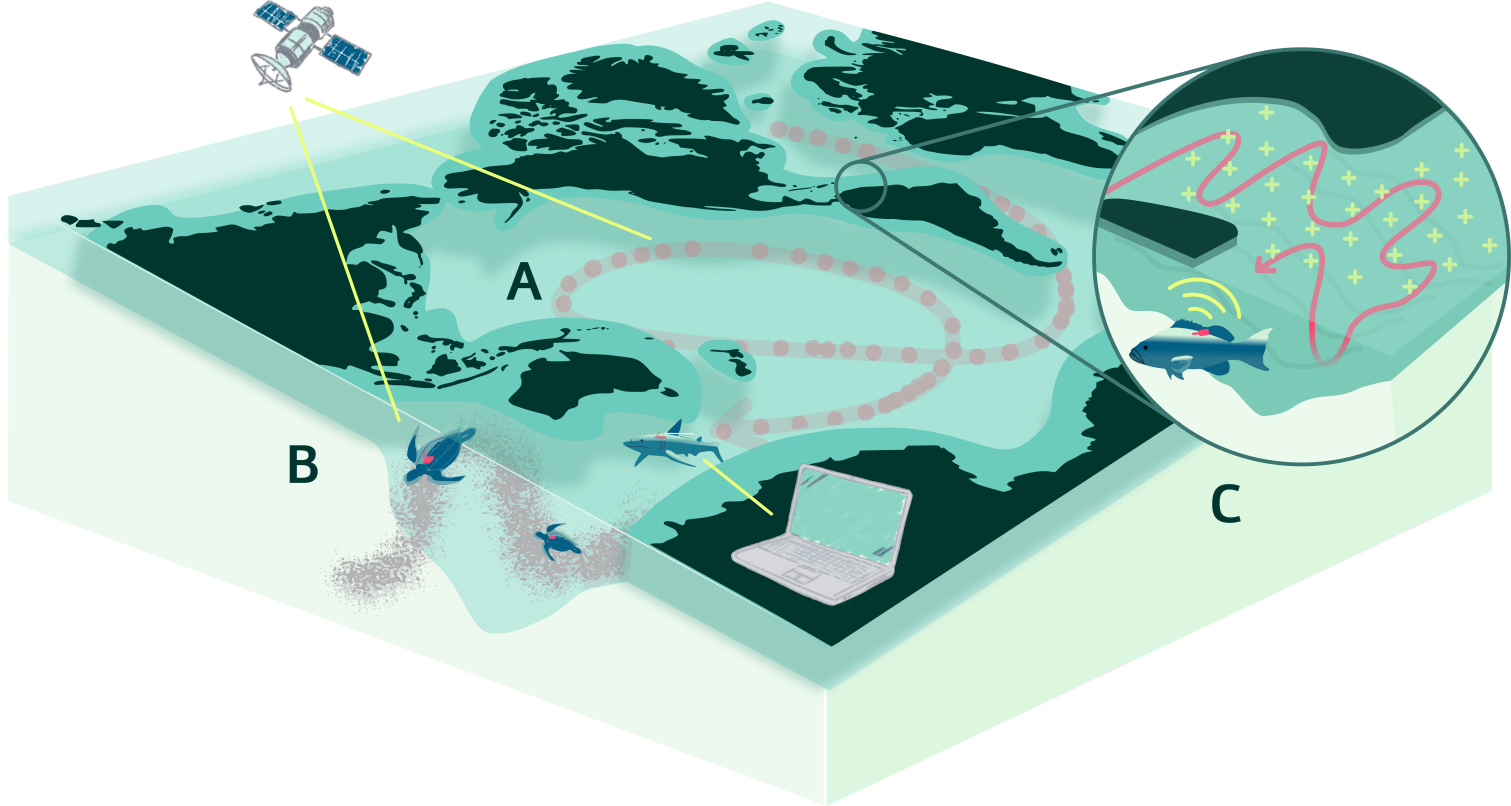
Examples of different tagging approaches and scales of resulting connectivity estimates, such as (A) transoceanic migrations of a shark and (B) vertical movements of turtles using satellite tags that transmit position and other data to satellites and/or data storage tags that need to be recovered to download the data. For finer scale and coastal movements, and for smaller bodied species, many researchers use biotelemetry methods (C), where the tag transmits a signal to a receiver array (yellow crosses). Artwork by Siegrid Mons (Siegrid Design), Scientific Graphic Designer.

The spatial extent of tagging methods varies by tag type, with mark–recapture limited to the release and recapture site. As a result, mark–recapture is heavily dependent on the distribution and effort of sampling or fisheries (Bolle et al., 2005), but engaging commercial and recreational fishers can help to increase coverage and recovery rates (Gillanders et al., 2001). The spatial extent of biologging tags tends to be unconstrained, reflecting the individual's full home range across all three dimensions (Figure 3). Indeed, electronic tags are one of the few methods available that can reliably estimate vertical connectivity (Shillinger et al., 2011). For acoustic biotelemetry, the horizontal spatial extent is restricted to the area encompassed by the receiver array, and coverage varies considerably by system (Ellis et al., 2019). Vertical connectivity can be estimated using acoustic pressure/depth sensor tags which transmit the depth estimate of the fish to the acoustic receivers in the array. For accurate triangulation of detections, robust testing of receiver range and synchronization of hydrophone clocks are essential prerequisites (reviewed in Kessel et al., 2014). However, when receiver spacing is relatively small and tagged animals are recorded by more than two receivers at a time, fine‐scale positioning is possible (Masmitja et al., 2020; Vigo et al., 2021). While biotelemetry studies can be restricted by the extent of their receiver array, data sharing can extend observations across ocean realms (Lilly et al., 2024; Lowerre‐Barbieri et al., 2021), with the evolution of large‐scale telemetry networks (Sturrock & Tanner, 2026a, 2026b), facilitated by entities such as the Ocean Tracking Network (OTN) (Iverson et al., 2019), being instrumental in overcoming the spatiotemporal constraints of this technique (Abecasis et al., 2018).

### Chemical markers

#### Methods

Chemical markers include elemental concentrations, stable isotope ratios, fatty acids, or contaminants derived from diet and environmental sources that are incorporated into animal tissues and structures (Campana & Thorrold, 2001; Trueman & St John Glew, 2019). Spatial variations in these markers may be driven by physicochemical variation in the water or biochemically mediated differences in lower trophic levels transferred through food webs (Trueman & St John Glew, 2019). Chemical markers can be used to retrospectively infer connectivity when information is known such as (1) the marker's environmental spatial and temporal variation, (2) how reliably organisms' tissues reflect environmental signals, and (3) how effectively we can measure variation in the marker (Elsdon et al., 2008). While chemical marker‐based approaches are inherently indirect and therefore generally have lower precision than direct tagging, their major advantage is that every organism is automatically “tagged” (Hobson, 1999). Critically, this allows for connectivity to be estimated for species or life stages too small to be externally marked or tagged. It is still a retrospective approach however and thus tends to be used on survivors, making it challenging to link spatial and demographic fates (Secor, 2015). Further, using chemical markers often requires upfront investment, such as extensive baselines and experimental work to understand the dynamics of marker incorporation for the given species and life‐history stage (Table 1).

##### Environmental signals and marker types

**FIGURE 4:**
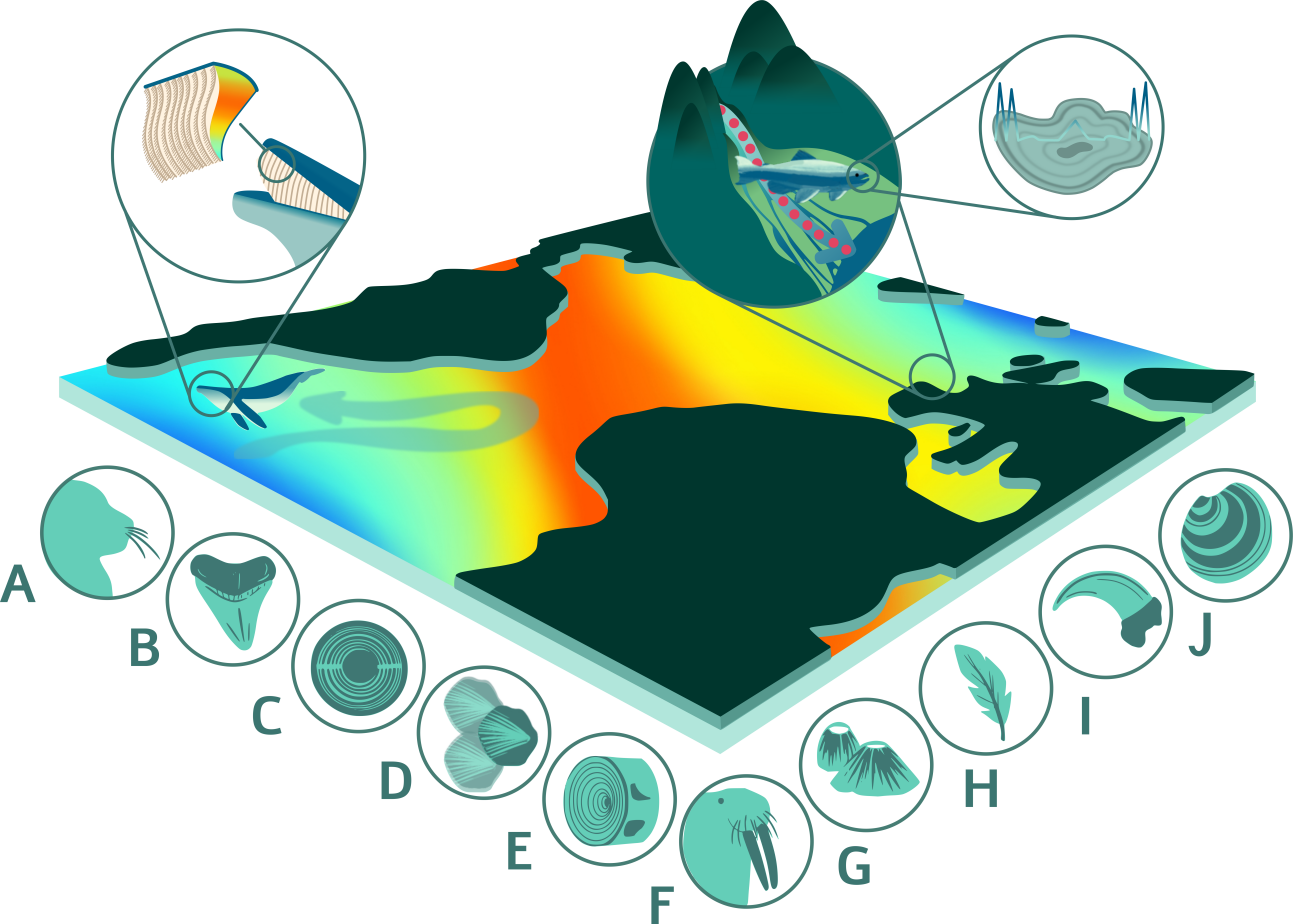
Examples of how chemical markers can be used to reconstruct transoceanic and fine‐scale movements, including a pole‐equator return migration of a whale using sequential isotope measurements in its baleen plate, and land–sea migration of salmon using otolith element concentrations. In addition to otoliths and baleen, A to G show examples of other archival structures: A—mammal whisker, B—shark tooth, C—fish eye lens, D—fish scale, E—shark vertebra, F—mammal teeth (e.g., walrus/narwhal tusk, sperm whale tooth), G—barnacle, H—bird feather, I—mammal claw, J—mollusk shell (note that cephalopods also have statoliths). Artwork by Siegrid Mons (Siegrid Design), Scientific Graphic Designer.

Chemical marker‐based geolocations are most effective when large physicochemical gradients exist, such as across the land–sea interface (Walther & Limburg, 2012) or ocean fronts (Ashford et al., 2007), as many elements and isotope ratios covary with salinity (e.g., Sr, Ba, and stable isotope ratios of strontium and oxygen, expressed as ^87^Sr/^86^Sr and δ^18^O values, respectively). In the open ocean, many elements are relatively homogenous (Sturrock et al., 2012); however, chemical gradients often exist in coastal ecosystems due to point source variations from upwelling (Woodson et al., 2013), river runoff (Mohan et al., 2018), or habitat heterogeneity (e.g., mangrove vs. seagrass sources, Kimirei et al., 2013). In open marine waters, isotopic and thermal gradients tend to be most useful for movement reconstruction, with biogeochemically induced variations in stable isotope compositions of carbon and nitrogen fixed by phytoplankton transferred through food webs (Hobson et al., 2010) and incorporation of oxygen isotopes into carbonate biominerals predictably related to temperature (Sakamoto et al., 2019). The physicochemical and biochemical mechanisms underpinning spatial variation in stable isotope ratios of C, N, and O are relatively well understood, and both statistical and mechanistic spatial models of isotopic variation across regional and global oceans have been developed (termed “isoscapes”, Trueman & St John Glew, 2019) (Figure 4).

Artificial chemical marking of otoliths, statoliths, and other calcified structures using elemental (e.g., SrCl_2_), isotopic (e.g., ^88^Sr/^86^Sr, ^138^Ba/^137^Ba), fluorescent (e.g., tetracycline), or colored (e.g., alizarin red) marks can also be used for mark–recapture studies (see above). Artificial marks may be applied by spiking the water (Munro et al., 2008) or diet (Woodcock & Walther, 2014), or through injection into the animal (Thorrold et al., 2006). Transgenerational marking, where gravid females are treated to impart chemical tags into calcified structures in their offspring, can allow MFC to be assessed across generations (Thorrold et al., 2006), while mass marking of eggs can be used to determine recruitment patterns of fish larvae (Jones et al., 2005). However, all spiking approaches require preliminary work to tailor the dose to the species, life stage, and tissue (Starrs et al., 2014), and marker toxicity needs consideration, given that the chemical may enter food webs and human diets.

##### Organism signals

Connectivity reconstructions often target incrementally grown or archival tissues that are metabolically inert or exhibit limited reworking (e.g., fish otoliths, scales, vertebrae, fish and cephalopod eye lenses, teeth, mammal tusks, bird feathers, mollusk shells and statoliths, turtle scutes and coral skeletons, Figure 4), with their inbuilt biochronology allowing the reconstruction of movements and environmental histories across an organism's lifetime (Hobson et al., 2010; Tzadik et al., 2017). Among these, carbonate biominerals such as otoliths, teeth, and shells tend to be more effective recorders of ambient water chemistry (e.g., Ba/Ca, Sr/Ca) and temperature (e.g., δ^18^O values), while proteinaceous structures such as eye lenses and baleen plates tend to better record biogeochemical variations in primary production via δ^13^C and δ^15^N values (Wallace et al., 2014). However, analysis of metabolically active matrices such as blood, lipid, muscle, and liver can also offer useful snapshots of trophic change and individual movement, so long as incorporation dynamics and tissue turnover rates are correctly interpreted (Herzka, 2005) (Figure 2).

An important first principle of inferring geolocation using chemical markers is that the tissue chemistry is predictably related to the environment. For stable isotopes, this is often the case, resulting in a variety of isoscape‐based applications (Cusa et al., 2021; Doubleday et al., 2022; St John Glew et al., 2018). However, relating the isotopic composition of a target organism to a reference isoscape may require consideration of the isotopic offsets between reference and target organism and the time taken to assimilate elements of interest into the tissue relative to its turnover rate. For applications hampered by limited isotopic variation in the environment, compound‐specific stable isotope analysis (CSIA) may increase spatial resolution, targeting compounds that can only be biosynthesised by microorganisms, which are therefore isotopically unaltered as they move through the food web. Essential amino acids and fatty acids are primary targets for CSIA‐based movement reconstruction, but to‐date, CSIA approaches are technically demanding and expensive, limiting widespread application (McMahon & Newsome, 2019; Twining et al., 2020). Carbonate biominerals have long been used as oxygen isotope thermometers, providing deep time records of the Earth's climate (McCrea, 1950). δ^18^O values in calcified structures can also be used as geo‐locators where patterns in water temperature and δ^18^O are known (Doubleday et al., 2022). Serial δ^18^O values measured in structures such as otoliths can be combined with models of water temperature to generate most likely movement tracks (Pearson et al., 2020; Sakamoto et al., 2019; Trueman & St John Glew, 2019). However, inferences may be limited by instrument or sampling resolution, a lack of spatially resolved water δ^18^O estimates, and species‐specific physiological influences on isotope dynamics (Darnaude et al., 2014; Morissette et al., 2023).

Element concentrations in animal tissues can also provide geographic information, but our understanding of ambient variation in ion availability and the influence of animal physiology on incorporation dynamics is less complete than for the isotopic approaches discussed above (Hüssy et al., 2021; Sturrock et al., 2015; Thomas et al., 2017). Consequently, most element‐based approaches to movement reconstruction draw on statistical comparisons among potential geo‐referenced sample populations (Elsdon et al., 2008; Reis‐Santos et al., 2023). More than 25 elements are routinely measured in calcified tissues using modern mass spectrometry approaches, providing potential opportunities to detect movement across isotopically or thermally homogeneous regions, especially in coastal waters where elemental variations are maximized due to riverine inputs (Gillanders & Kingsford, 1996).

##### Analysis methods

The success of chemical markers as geo‐locators also depends on the analytical approach and the instrument type and its detection limits. Bulk approaches such as whole dissolved otoliths (typically analyzed for element concentrations using solution‐based inductively coupled plasma‐mass spectrometry [ICP‐MS] or atomic emission spectroscopy [ICP‐AES]) or soft tissues (typically analyzed for stable isotope ratios using isotope ratio mass spectrometry [IRMS]) are faster and cheaper, and typically used to describe population‐specific signatures and track their frequency during brief mixing periods (Campana et al., 2000; Witteveen et al., 2009). However, bulk approaches only provide a single value, where the time period is determined by the tissue's growing period and turnover rate, potentially masking spatial shifts if there is a mismatch between movement rate and signal integration. In contrast, microsampling of archival tissues using dissection (e.g., feathers and eye lenses, Hobson et al., 2010; Wallace et al., 2014), hand drill or computer‐guided micromill, or probes such as laser, electron, or ion beams (Hanson et al., 2010), allows specific time periods to be targeted or lifetime profiles be constructed, sometimes achieving subweekly resolution, depending on tissue growth rate and the amount of material required (Sakamoto et al., 2019). Alternatively, imaging methods (e.g., laser ablation time‐of‐flight ICP‐MS and nondestructive methods such as the synchrotron and micro‐PIXE) are gaining popularity to map variations in chemical markers across the entire structure, allowing extraction of time series data across multiple axes (Limburg & Elfman, 2017).

#### Data analysis approaches

Inferring location and MFC patterns from chemical markers generally draws on three broad data analysis approaches, depending on the study design and reference data available. Studies typically aim to (1) assign individuals to most likely locations among discrete, identified potential sources (nominal prediction), (2) identify likely geographic origin across continuous spatial surfaces, or (3) identify likely clusters of individuals with no direct spatial reference. In addition, to understand the drivers of movement, linear or additive mixed‐effects models can link profile data across an archival structure with abiotic and biotic variables while dealing with temporal autocorrelation in the data (Ng et al., 2025; Sturrock et al., 2015).

For option (1), trace element data in otoliths have long been used to assign individuals to likely discrete reference populations using a range of supervised statistical methods such as random forest, discriminant analysis, and canonical analysis of principal coordinates (CAP). Maximum likelihood estimation (MLE) (Rooker et al., 2014; Tanner et al., 2013) and Bayesian mixture models (Macdonald et al., 2021; Munch & Clarke, 2008) can also be used to estimate the relative contribution of different sources. These methods all require a suitable reference library to assign individuals of unknown origin (Mercier et al., 2011). Given the reasons outlined above, for element‐based applications, reference libraries tend to comprise tissues from individuals of the same species and life stage, collected from each putative source. Relying on a set number of reference groups may induce bias, as it is rare for all potential reference groups to be characterized; however, Bayesian techniques can help to account for unsampled groups (Neubauer et al., 2013).

For option (2), chemical tracers can be used to identify most likely location within a continuous spatial area, so long as a statistical model of chemical tracer variation is available, that is, an appropriate isoscape (Trueman & St John Glew, 2019) or chemoscape (Burns et al., 2020). R‐based statistical packages such as *AssignR* can assist isoscape‐based statistical assignment of organisms to likely location (Ma et al., 2020). It is also possible to combine (1) and (2), for example, by integrating machine learning multi‐model ensemble classifiers using Bayesian model averaging (nominal) with continuous surface isoscapes (Arai et al., 2023).

For option (3), unsupervised statistical methods (e.g., clustering, non‐metric multidimensional scaling or Bayesian mixture models) identify potential groups in the data based on variation in chemical signatures without providing prior information, such as geographical location. This is often used to infer potential fish spawning or nursery groups based on otolith core or near‐core signatures (Ashford et al., 2008; Reis‐Santos et al., 2018).

Finally, for chemical profiles, time series clustering (Shima & Swearer, 2009), dynamic time warping (Arai et al., 2026; Hegg & Kennedy, 2021), and change point analysis (Reis‐Santos et al., 2025; Sabetian et al., 2021) can infer lifetime movements and migratory contingents.

#### Temporal and spatial scales

For incrementally grown and metabolically inert structures such as otoliths, eye lenses, and shells, chemical markers can theoretically decipher movements over any part of that individual's lifetime, but the temporal resolution usually depends on the structure's growth rate and the sensitivity of the analytical method (Elsdon et al., 2008) (Figure 2). The temporal scales achievable using chemical markers can also extend well beyond the life of an individual to reveal historical or paleontological patterns in MFC, as many calcified structures are archived in fisheries institutes and museums, or remain well preserved in archaeological middens (Disspain et al., 2016).

Chemical markers in archival tissues can unlock connectivity from a few kilometers (McMahon et al., 2012; Phillis et al., 2018) to across ocean basins (Ashford & Jones, 2007; Rooker et al., 2014), depending on the scale of the environmental gradient and its successful integration into the animal's tissue (Figures 2 and 4). The spatial scale of connectivity estimates derived from spiking approaches are—like all mark–recapture approaches (see above)—largely dependent on sampling coverage and recovery rate, and thus tend to be better suited to species with high site fidelity or homing behavior (Almany et al., 2007), and where there are easy opportunities for mass marking (e.g., stocking and aquaculture) (Munro et al., 2008).

Chemical markers in soft tissues generally provide coarser spatiotemporal resolution; however, advances in isoscape modeling, methods such as isotopic clocks, and habitat‐specific baselines, are likely to further refine such methods (e.g., Besnard et al., 2023; Shipley et al., 2021). Finally, integrating markers across multiple tissue types within individuals can improve the resolution of connectivity estimates and provide additional insights into trophic pathways (Toledo et al., 2020) and contaminant exposure histories (Dietz et al., 2021).

### Genetics

#### Methods

Genetic approaches to connectivity estimation depend on the assumption that exchange of individuals (or the lack thereof) among populations leaves a genetic signature that can be used to infer historical and contemporary connectivity patterns (Hellberg, 2009). Most methods for estimating genetic connectivity require spatial variation in the genetic composition of local populations (subpopulations or demes), with parentage‐based analysis being the exception. This represents a challenge for population genetic methods because the high dispersal abilities and other attributes of marine species (most notably, large population sizes) limit genetic differentiation among populations. As a result, commonly employed genetic approaches for estimating population connectivity are most powerful at revealing the absence of gene flow between areas (Lowe & Allendorf, 2010; Waples & Gaggiotti, 2006) (Table 1).

**FIGURE 5:**
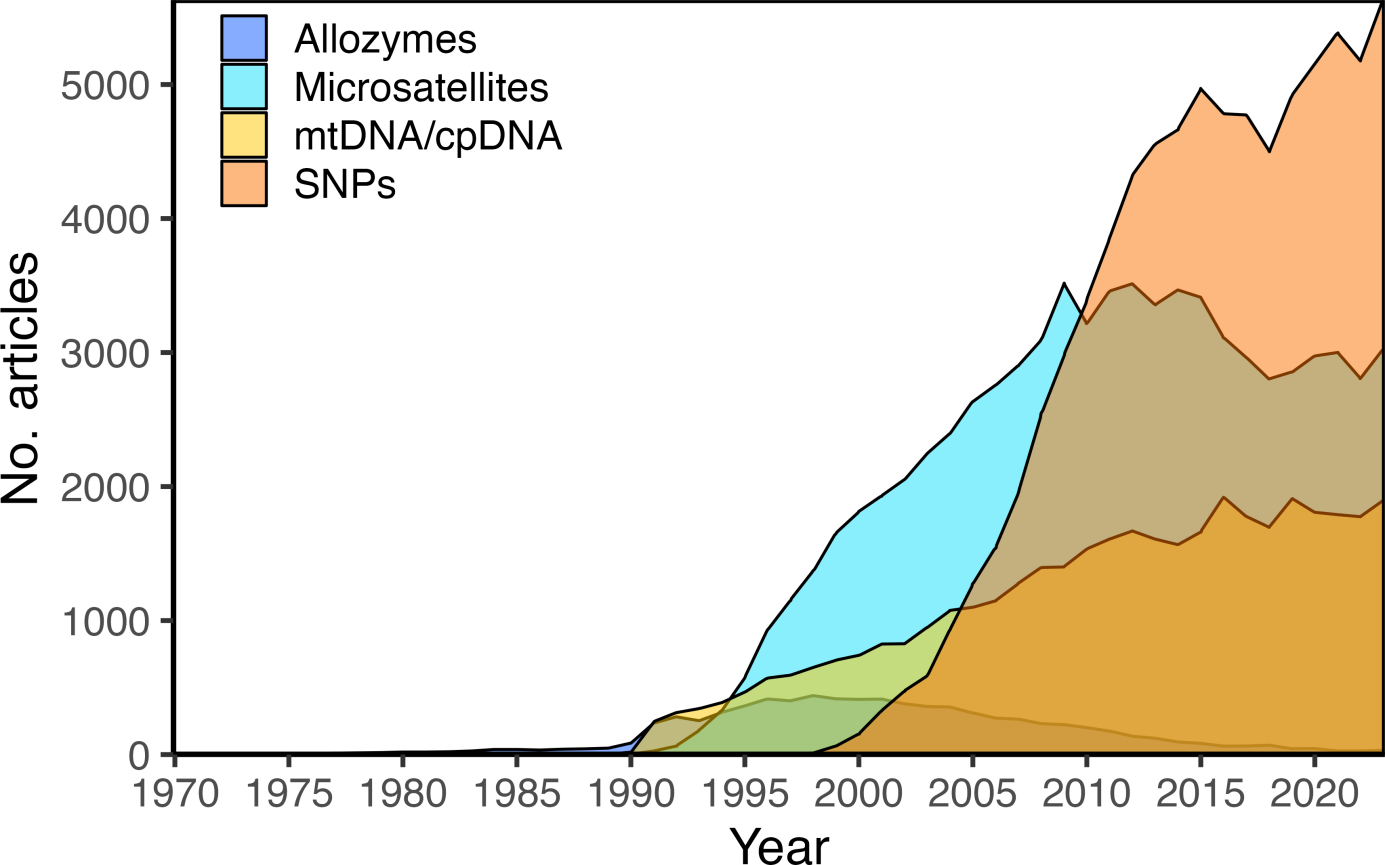
Temporal changes in the popularity of genetic markers. Four Web of Science Topic searches (articles only) were conducted on April 4, 2024 using the following terms: “Allozyme,” “Microsatellite,” “mitochondrial or chloroplast (mtDNA) OR chloroplast DNA (cpDNA),” and “single‐nucleotide polymorphisms (SNPs).” Total number of articles identified were 7998, 39,289, 75,587, and 78,924, respectively, separated by publication year (2023 and 2024 merged). The total number of articles found between 1970 and 1985 was 690 (89.5% allozymes, 10% mtDNA or cpDNA, 0.5% microsatellites). These searches illustrate global shifts in genetic marker usage over time rather than trends limited to marine systems.

Where connections are minimal, the extensive literature of phylogeography and population genetics demonstrates a variety of genetic tools that can identify historical barriers to dispersal, both among (Patarnello et al., 2007) and within basins (Costantini et al., 2018). Persistent dispersal barriers can be readily identified using even relatively low numbers of loci and a range of genetic markers. For instance, phylogeographic studies based on single locus mitochondrial or chloroplast loci all the way through to multilocus population genetic studies based on a handful of nuclear markers such as microsatellites (Figure 5) will often detect persistent dispersal barriers. However, genomic investigations that draw information from thousands of loci (whether it be single‐nucleotide polymorphisms [SNPs], microsatellites, or other genetic markers) are steadily replacing studies relying on one or few loci (Figure 5, also Schlötterer, 2004). Through representative sampling of loci at the whole genome scale, genomics provides enhanced power to detect weaker restrictions to gene flow and enables insights into more recent timeframes than investigations based on few loci, thereby bringing enhanced power to connectivity estimation (Nikolic et al., 2020).

#### Data analysis approaches

**FIGURE 6:**
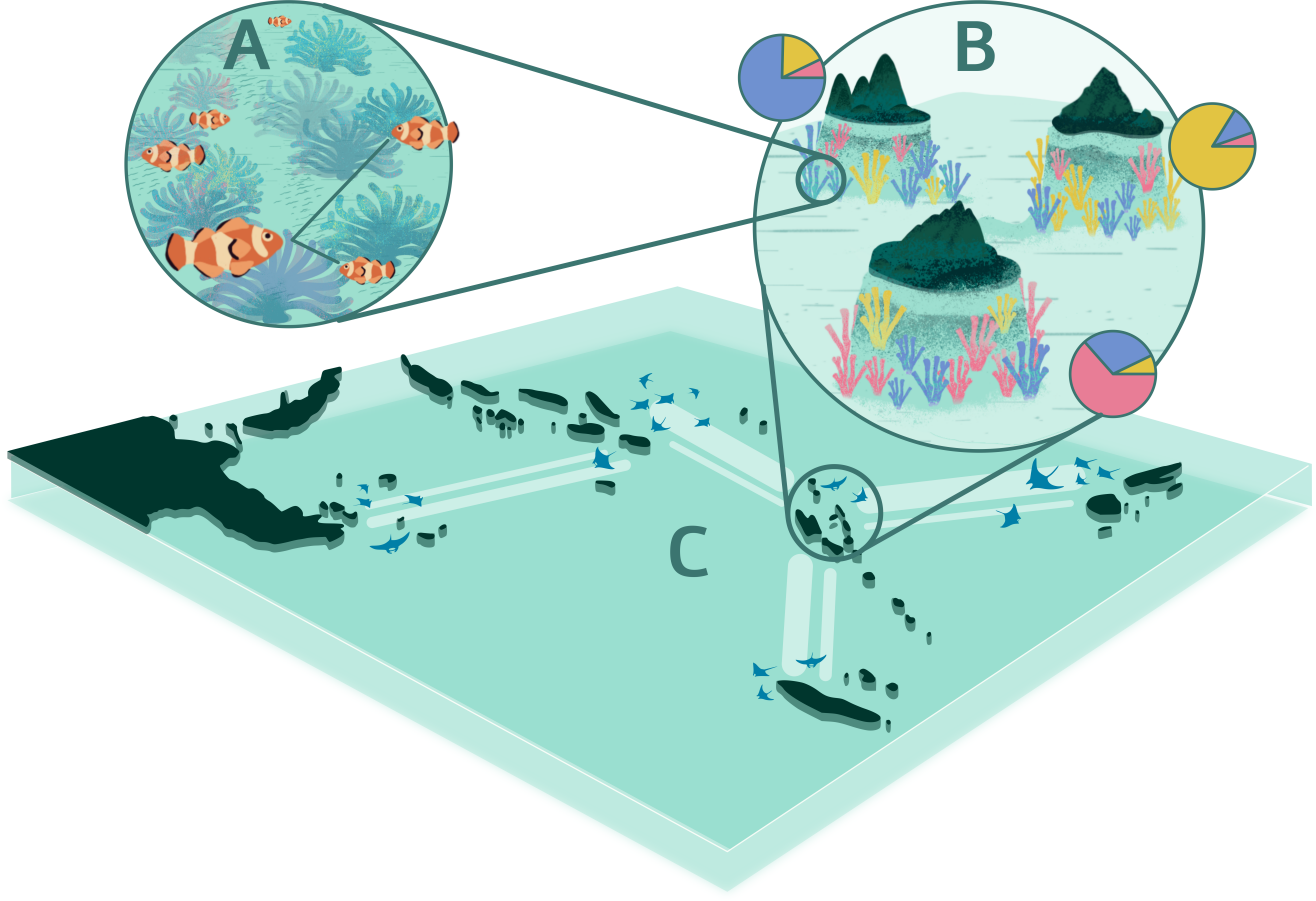
Three main approaches to estimating marine functional connectivity (MFC) using genetic markers from fine (tens of kilometers) to large (thousands of kilometers) spatial scales: (A) parentage analysis—example showing clownfish around a single island, (B) assignment tests—example showing coral populations of a single species around three islands where their color and the pie charts show proportional assignment to distinct genetic groups, and (C) coalescent and diffusion models—example showing manta rays that exhibit different levels of genetic exchange and isolation across a large island chain. Artwork by Siegrid Mons (Siegrid Design), Scientific Graphic Designer.

Three main methods are commonly used to infer connectivity: (1) parentage, (2) assignment tests, and (3) coalescent and diffusion models (also referred to as model‐based demographic inference) (Figure 6). These methods all require multilocus data and are therefore well suited to genomic approaches. Parentage analysis uses multilocus genotypes to probabilistically identify offspring and parents (and sometimes siblings and grandparents; reviewed in Flanagan & Jones, 2019). If the relative spatial positions of individuals are known, then dispersal distances and directions can be directly calculated. Parentage approaches have been used extensively for reef fishes with small home ranges such as clown fishes (Planes et al., 2009; Salles et al., 2020), but also for fishes that disperse up to 30 km (Harrison et al., 2012) and some invertebrates (Dubé et al., 2020). Parentage methods have the advantage of being conceptually simple and can measure recent connections (1–2 generations) in all directions surveyed, but rely on intensive sampling and therefore are of limited utility for taxa that disperse long distances and/or show large population size, two features very common in the marine realm (Gagnaire et al., 2015). Assignment tests such as *STRUCTURE* (Pritchard et al., 2000) and *ADMIXTURE* (Alexander et al., 2009) are better suited to sampling over longer distances and can detect immigrants (or individuals of immigrant ancestry). The same underlying principles have been used to derive methods specifically aimed at estimating the “migration matrix” (full set of pairwise migration rates between populations) using programs such as *BayesAss* (Wilson & Rannala, 2003) and Bayesian inference of immigration rates (*BIMr*) (Faubet & Gaggiotti, 2008). Assignment methods are convenient in that sampling can be spatially extensive and include many populations; however, the power to detect immigrants relies on populations being sufficiently differentiated to discriminate between migrant and local individuals, and therefore, connections must be infrequent (Christie et al., 2017). However, many of the implemented methodologies (e.g., BIMr; Faubet & Gaggiotti, 2008) are computationally expensive and can only accommodate a limited number of subpopulations and loci, and thus warrant future methodological developments.

Parentage and assignment tests rely on genotypes of individuals and—while they can be undertaken with microsatellites—they gain more power from including more loci. Both methods require an appropriate characterization of the genetic composition of the populations from which migrants originate. This requirement is similar to the need to obtain representative baseline samples when using chemical markers (see above). In the case of parentage analysis, this requires genetic sampling of a large fraction of the parental (adult) population. For assignment tests, this requires sampling of all or most extant populations; otherwise, migrants may be wrongly assigned to a population simply because it is genetically similar to an unsampled population, a so‐called “ghost population” (reviewed in Christie et al., 2017).

Coalescent and diffusion models require genomic scale data and use information from gene trees and/or allele frequency spectra (the proportions of alleles of different frequency classes). These methods evaluate the probability of specific past scenarios describing relationships among few (most often two) populations. These scenarios typically include: (1) no connectivity (isolation, or divergence only), (2) continuous low‐level connectivity over time (isolation‐migration), and (3) intermittent connectivity through time (secondary contact). Popular software implementing coalescent and diffusion models include *IMa* (Hey et al., 2018), *dadi* (Gutenkunst et al., 2009), and *fastsimcoal* (Excoffier et al., 2013). Because recent isolation and ongoing migration connections (where migration is here defined as the probability of an individual dispersing from a source population and contributing genetic material to the receiving population) lead to similar genetic patterns (Gagnaire et al., 2015), a major strength of coalescent and diffusion modeling is the capacity to jointly estimate isolation and migration. Additionally, migration directionality and strength can be estimated (Figure 6). For example, recent demographic inference based on thousands of SNPs uncovered substantial gene flow of Atlantic bluefin tuna from Mediterranean stocks into western Atlantic stocks (Díaz‐Arce et al., 2024). However, such analyses are restricted to few populations and are computationally intensive and complex. They also include many parameters that all need to be estimated (e.g., effective population sizes for all demes, migration timing and rates, bottleneck strength and timing, population growth rates), introducing identifiability issues, as different combinations of parameter values can lead to the same outcome.

While genetic methods are unable to detect signals when connectivity is high (Table 1), several new methods are starting to be applied to marine taxa that appear promising, based on the fine‐grained analysis of spatial gradients of differentiation (Gazulla et al., 2021; Jeannot et al., 2024). For instance, dispersal estimation based on isolation by distance theory (Rousset, 2000, 1997) has been applied to several studies of reef fishes, which support a general conclusion that most larval dispersal occurs on short geographic scales (generally less than 200 km and less than 30 km for some species) (Naaykens & D'Aloia, 2022; Pinsky et al., 2017; Puebla et al., 2009). Seascape genetics (see *Method integration*: *Genetic approaches combined with numerical modeling*) correlate patterns of genetic differentiation between populations with competing spatial and environmental predictors to infer which past or present seascape features best predict observed connectivity patterns (Assis et al., 2018; Riginos et al., 2011; Selkoe et al., 2008). These approaches bear the implicit assumption that detected correlations are relevant to contemporary connectivity patterns and that genetic similarity reflects gene flow and not recent divergence; for example, populations that share recent ancestry such as from a range expansion can be genetically similar even if there is no contemporary gene flow. In general, combining inferences from genetics with other information such as historical records of habitat distribution and modeled trajectories based on biophysical modeling increases confidence (see *Method integration*: *Genetic approaches combined with numerical modeling*). Furthermore, new genomic methods draw upon information in long tracts of chromosomes (Leitwein et al., 2020) to make inferences about timing and directionality of gene flow, for example, identifying past movement of the European sea bass into the Mediterranean (Duranton et al., 2018). Finally, new approaches based on patterns of kin structure and life‐history information to estimate stock abundance may lead to new methods to estimate connectivity. For example, close‐kin mark–recapture (CKMR; Bravington et al., 2016), while primarily designed to estimate stock sizes or biomass through datasets based on large‐scale and high‐density sampling, may also allow estimating minimum parent‐offspring dispersal distance, albeit further research is required to confirm its applicability.

#### Temporal and spatial scales

The genetic composition of populations is shaped by long‐term processes spanning both evolutionary and ecological time scales (Figure 2), and it is difficult to distinguish between connection signatures from the distant past and those that occurred recently. Furthermore, there can be a long time lag between a demographic event (such as population fission and bottlenecks) and its effect on the genetic structure of populations (Bailleul et al., 2018). In contrast, most connectivity studies, especially those seeking to use the data for ecological inference, are interested in shorter time scales such as a few generations. Because dispersal abilities can vary greatly among marine taxa, the relevant spatial scale for recent connectivity inference will depend on the species' biology, such as its reproductive strategy, PLD, and behavior. For example, coral genetic structuring has emerged at distances of thousands of metres across the Red Sea (Rachmilovitz et al., 2024) to <1 m within caves (Costantini et al., 2018).

The genetic methods best suited to detecting recent connections are parentage analysis (1–2 generations) and assignment tests (1–3 generations). However, at small spatial scales that allow genetic equilibrium to be rapidly attained, isolation by distance provides reliable estimates of parent‐offspring distances (Rousset, 2000), leading to connectivity estimates congruent with parentage analyses (Pinsky et al., 2017). By definition, parentage methods are conducted over relatively short distances, namely, the distances over which single generational dispersal occurs, and do not require migration‐genetic drift equilibrium. Isolation by distance also provides estimates of single generational dispersal (i.e., parent‐offspring distance) but assumes migration‐genetic drift equilibrium, so such estimates represent averages over many generations. Assignment tests are better suited to larger spatial scales, as they rely on inherent differentiation between populations. Demographic inferences using coalescent and diffusion models and haplotype tract methods are more appropriate to long time scales and greater spatial distances.

### Numerical modeling

#### Methods

**FIGURE 7:**
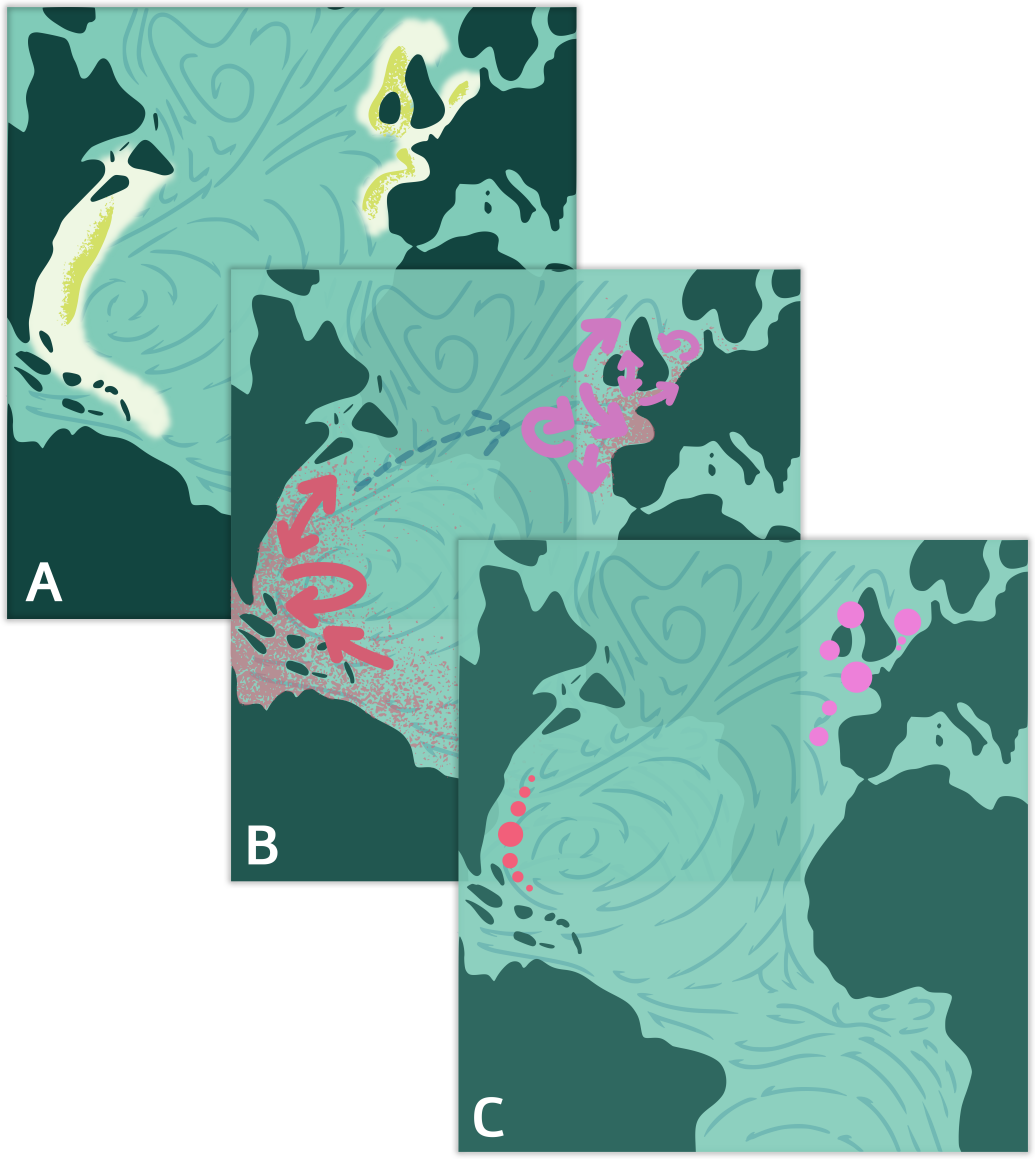
Examples of numerical model outputs used to estimate or support marine functional connectivity (MFC): (A) Species distribution models (SDM) to predict where species are most likely to be present (green/cream shading display a species' suitable habitat), based on observed presence/absence data. (B) Biophysical models use the direction and strength of currents (thin arrows) together with biological information (e.g., pelagic larval duration [PLD]) to simulate the dispersal of larvae (pink dots) and estimate potential MFC (thick pink/purple arrows) among suitable habitat areas. The dashed arrow represents an example of where oceanographic connectivity is decoupled from MFC due to the PLD for this species being too short for the eggs and larvae to travel this distance. (C) Spatially explicit population/community dynamical models integrate MFC estimates for different life stages over time and space to predict population density/diversity spatial distribution (pink/purple circles) within the species habitat. In all layers, the thin blue arrows indicate the major oceanic currents. Artwork by Siegrid Mons (Siegrid Design), Scientific Graphic Designer.

There are three numerical modeling approaches that are central to MFC research: (1) species distribution models (SDMs), (2) biophysical modeling, and (3) spatially explicit dynamical modeling (Figure 7). A key strength of these models is that they can be used in hindcast and forecast mode, the latter facilitating prediction under a range of future scenarios (e.g., climate change, management options) (Table 1 and see *Method integration*: *Genetic approaches combined with numerical modeling*).

SDMs use environmental layers and occurrence records of the focal species to predict its distribution across space (González‐Irusta et al., 2015). The results of these models are represented as maps of predicted suitable habitat for the species, allowing the identification of discontinuities that may act as natural barriers to functional connectivity (Sundblad et al., 2011). While SDMs, Ecological Niche Models, and Habitat Suitability Models are based on subtly different concepts, they are broadly synonymous in their application (Peterson & Soberón, 2012). SDMs can be fitted using presence, presence/absence, or abundance data. Their outputs do not reveal the direction of connectivity fluxes but are useful for predicting the influence of alternative climate scenarios or management actions (e.g., fishing bans) on population dynamics and potential MFC patterns.

Biophysical connectivity models are the only modeling approach that produce mechanistically derived estimates of directional MFC fluxes between release and destination locations, synthesized into a connectivity matrix. They simulate organism trajectories, integrating physics (dynamical equations in time and space) with biology (biological constraints such as PLD and swimming ability). They typically use hydrodynamical and biogeochemical forcings (mainly modeled data but sometimes surface current data derived from direct satellite observations) to predict organism transport. Larval transport can be predicted using Lagrangian particle tracking (Johnson & Hess, 1990) and Eulerian advection–diffusion modeling (Ellien et al., 2000). The Lagrangian approach, which allows tracking both forwards and backwards in time, is now the most commonly used due to its individual‐based perspective that allows the inclusion of factors affecting motility and survival such as ontogenetic development (Lett et al., 2008; Paris et al., 2013) and dynamic energy budgets (López et al., 2013). A key strength of biophysical dispersal models is that they can provide information on connectivity features, including directionality, when outputs are analyzed as transfer rates between locations. The accuracy of biophysical connectivity models depends on the level of integration of biological parameters (eggs/larval/population level) describing organism morphology, behavior, and ecological traits, with physical parameters, while computing organism dispersal (distance and direction). Missing or limited information on species‐specific traits (e.g., spawning timing and duration, PLD) or the lack of direct validation data (e.g., three‐dimensional trajectories) can lower the accuracy and precision of connectivity estimates.

Spatially explicit dynamical models upscale and integrate connectivity estimates for different life stages over time (species' life cycle) and space (species' habitat/niche) to produce spatiotemporal predictions of population dynamics (Carson et al., 2011; Watson et al., 2012) or diversity dynamics (Moritz et al., 2009). Spatially explicit models of population dynamics require both connectivity estimates and life‐history traits (e.g., longevity, age at sexual maturity, fecundity, life stage specific survival rates), and spatially explicit models of community dynamics additionally require estimates of the strength of species' interactions. Hereinafter, for brevity, we refer to spatially explicit population dynamical models as metapopulation models and spatially explicit community dynamical models as metacommunity models. In doing so, we follow the broader usage common in the marine literature, in which metapopulations are understood as spatially structured populations connected by dispersal or demographic exchange rather than being restricted to systems with recurrent local extinctions (Hanski & Gaggiotti, 2004; Kritzer & Sale, 2004).

#### Data analysis approaches

SDMs are correlative approaches using environmental layers in a GIS format (usually a raster) and a species' presence records to predict its distribution (González‐Irusta et al., 2015).

Biophysical models typically rely on hydrodynamical models and individual motility traits to predict connectivity matrices and dispersal kernels. For example, larval swimming behavior and PLD can have significant influence on dispersal distance (Barbut et al., 2019; Bode et al., 2019), with implications for population persistence (Guizien et al., 2012). Algebraic analysis methods have been applied to identify functionally connected networks, with particular emphasis on the graph theory approach (Costa et al., 2017; Jacobi & Jonsson, 2011).

Metapopulation and metacommunity models upscale the spatiotemporal dynamics of ocean physics and biogeochemistry to dynamical outputs of its biota. Metapopulation models can produce maps of population density (Bendtsen & Hansen, 2013; Guizien et al., 2014) and population genetics (Jollivet et al., 1999; Padrón & Guizien, 2016). Finally, metacommunity models combine multiple spatially explicit metapopulation models with species interactions, and while scarce, can also map community diversity (Moritz, 2010).

The three modeling approaches can also be combined to improve connectivity estimates and species distribution predictions (Cecino et al., 2021; Foltête et al., 2012; Inglis et al., 2006). SDMs can inform biophysical models by predicting species presence in areas of interest, for example, for predicting connectivity between MPAs (Gallego et al., 2017). Conversely, biophysical models can inform SDMs by incorporating connectivity data as an explanatory variable (Cecino et al., 2021; Inglis et al., 2006). Finally, spatially explicit dynamical models rely on habitat suitability and migration data, which can be derived from SDMs and biophysical models, respectively (Guizien et al., 2014; Theuerkauf et al., 2021).

#### Temporal and spatial scales

Across models, the resolution of MFC estimates tends to be limited by the spatial resolution of environmental descriptors and ocean flow, particularly for species associated with patchy habitats such as sessile species attached to hard substrates (Blouet et al., 2024; Sciascia et al., 2022).

SDMs can be developed for a wide range of spatial scales, depending on the size of the studied area, the resolution of the environmental information available for that area and the geolocation error associated with the presence records. Depending on this, SDMs can use grid cells of a few metres (de la Torriente et al., 2019) to several kilometers (González‐Irusta & Wright, 2017). SDMs do not always include a temporal scale, and they are often developed using presence records across different periods, especially for sessile fauna. However, when necessary, SDMs can include different periods, limited to the availability of environmental layers with equal periodicity and covering the study area (e.g., Arronte et al., 2022; González‐Irusta & Wright, 2017).

Most biophysical models concentrate on forecasting the dispersal of marine organisms during their early, drifting larval phase over days to months, and spatial scales ranging from 100 m to 1000 km. The release period for larvae can span from a single breeding season to longer term scenarios of up to 100 years to capture variability and long‐term trends. Relatively few models address the dispersal of juveniles and adults (Tu et al., 2012), which typically involve more active behavior than larval dispersal. The focus on early life stages is due to the critical role that larval dispersal plays in shaping population connectivity, genetic diversity, and the overall structure of marine populations. However, a comprehensive understanding of marine population dynamics and connectivity requires integrating models across all life stages.

Spatially explicit dynamical models exhibit temporal scales typically from about 1 to 1000 years, and spatial scales of about 10–1000 km (Kjelland et al., 2015; Tuck & Possingham, 2000) (Figure 2). In the context of these models, a one‐year time step might capture annual cycles, breeding seasons, and short‐term population dynamics such as seasonal migrations and fluctuations in population size. Longer temporal scales, spanning centuries, are used to understand evolutionary processes, long‐term species' persistence, habitat changes, and the impact of rare but significant events such as climate change, habitat fragmentation, and large‐scale disturbances (e.g., fires, hurricanes). At local scales (~10 km), spatially explicit dynamical models might focus on specific habitat patches, local interactions among individuals or species, and short‐range dispersal, providing insights into fine‐scale ecological dynamics and local population viability. Conversely, at larger spatial scales (up to 1000 km), they consider broader seascape connectivity, long‐range dispersal events, regional migration patterns, and interactions across multiple habitat patches, which is crucial for species with large home ranges or that rely on multiple habitats over their life cycle.

## METHOD INTEGRATION

Method integration can overcome many of the limitations of individual methods and increase the spatiotemporal resolution, accuracy, and reliability of resulting MFC estimates (Figure 2). Several approaches have been used to combine information from different methods. Most commonly, data obtained from one method are used to support inference drawn from another (Delerue‐Ricard et al., 2019; Hanson et al., 2022; Jahnke et al., 2018; Riginos et al., 2019; Rogers et al., 2021; Wright et al., 2018). Alternatively, outputs can be used to infer an unknown parameter such as PLD (Padrón et al., 2018), or to infer connectivity at different spatiotemporal scales to address different questions (e.g., Abaunza et al., 2008; Papetti et al., 2013). Partial integration can be achieved by inputting individual probabilities of belonging to a group (in these examples, using genetics) into a classification model along with other numerical population markers (Higgins et al., 2010; Tanner et al., 2014). Arguably, the most powerful approaches integrate the raw data from two or more methods within a single statistical analysis, which often involves combining continuous (e.g., otolith chemistry) with categorical (e.g., genetic) data (Brophy et al., 2020; Smith & Campana, 2010; Zhang et al., 2021). Such fully integrative approaches use flexible modeling methods that can deal with multidimensional and diverse datasets, such as Bayesian frameworks or supervised learning techniques (e.g., support vector machine, random forest) (Brophy et al., 2020; TinHan et al., 2020). A comprehensive supervised machine learning framework for integrating genetic data with other population markers (e.g., chemical markers, morphological markers) is provided by the *assignPOP* R package (Chen et al., 2018). This package includes procedures to independently cross‐validate assignment accuracy of baseline data (individuals of known origin) and offers several methods for predictive assignment of individuals of unknown origin, including discriminant analysis, support vector machine, naïve Bayes, decision trees, and random forest.

To date, most integrative studies have combined information from two methods; usually genetic markers with one other source of information. However, Gaggiotti (2017) presented a fully integrative framework which can combine multiple data sources, with the example presented combining individual genotypes with connectivity matrices based on otolith chemistry data and biophysical modeling (but it could have also included abundance or tagging data). The data inputs are combined within a hierarchical Bayesian model, whilst incorporating the uncertainty associated with each approach, paving the way towards complete integration of all available connectivity information. In addition to flexible statistical frameworks that can deal with complex multidimensional datasets, full method integration requires multidisciplinary collaboration and coordination to ensure that, where possible, multiple techniques are applied to the same individuals (Abaunza et al., 2008, 2014). In subsequent sections, we highlight specific examples of different methodological combinations, identifying the advantages of integration, the additional methods required to support integration, and future innovations.

### Genetic approaches combined with numerical modeling

The most common integrative approaches combine genetics with numerical modeling. In particular, the burgeoning field of seascape genetics combines measures of genetic structure with spatially resolved environmental data to identify features related to both neutral and adaptive genetic variation (reviewed in Liggins et al., 2013; Selkoe et al., 2008). Seascape genetics provide a powerful tool to understand the circulation patterns and dispersal barriers leading to population connectivity and genetic differentiation (Jahnke & Jonsson, 2022). As advances in high‐throughput (next generation) sequencing have enabled detailed investigation of genomic diversity and the identification of candidate loci under selection, seascape genomics has been used to infer the environmental mechanisms underpinning allelic variation across many loci (Riginos et al., 2016) and genetic adaptation (e.g., Coscia et al., 2020; Diopere et al., 2018; Sjöqvist et al., 2015). A key advantage of seascape genomics is the opportunity to couple genome‐wide screening techniques with high‐resolution biophysical models that offer both fore‐ and hindcasting abilities.

A common approach in seascape genetics/genomics is to use a biophysical model to generate a connectivity matrix, which captures the probability of dispersal between each location, and to compare this to pairwise measures of genetic differentiation such as *F*_ST_ or estimates of gene flow (e.g., Jahnke et al., 2018; Riginos et al., 2019). Network analysis of the connectivity matrix can also be used to derive connectivity metrics for each site, which can be related to site‐specific allele frequencies using regression or multivariate approaches (Diopere et al., 2018; Munguia‐Vega et al., 2013). A biophysical model connectivity matrix can also be paired with a genetic simulation model to forecast changes in allele frequencies over multiple generations, which is particularly useful for detecting stepping stone connectivity (where dispersal to a habitat patch within one generation facilitates dispersal to more isolated patches in subsequent generations) (Padrón & Guizien, 2016). Independent of the analysis approach used, congruence of biophysical and genetic connectivity measures indicate that ocean circulation patterns likely played a role in shaping the observed population structure (e.g., Jahnke et al., 2017), while disagreement suggests that other mechanisms, such as the migration of adults, are involved (e.g., Valencia et al., 2021).

The power of these approaches can be strengthened by refining and standardizing current methods, considering spatial‐autocorrelation patterns, and incorporating demographic modeling (Jahnke & Jonsson, 2022). Achieving its full potential demands multidisciplinary teams and—to resolve the complex processes underlying genetic structuring and connectivity in marine systems—increased availability of oceanographic data with sufficient spatiotemporal resolution, particularly in nearshore areas (Liggins et al., 2013; Riginos et al., 2016; Selkoe et al., 2016). As the field develops, the incorporation of connectivity, population dynamics, and evolutionary processes into spatial management and conservation will be greatly aided by implementing fully integrative frameworks such as those described by Baltazar‐Soares et al. (2018) and Gaggiotti (2017).

### Genetic approaches combined with chemical markers

Genetic data are often combined with chemical markers in organism tissues to integrate connectivity information over evolutionary and demographic time frames, respectively. These two types of data have been combined by comparing connectivity patterns derived independently by each method (Papetti et al., 2013; Taillebois et al., 2017) and using fully integrated modeling approaches (Brophy et al., 2020; Smith & Campana, 2010; TinHan et al., 2020). While many studies pair genetics and otolith chemical markers to study fish stock structure (Cadrin & Secor, 2009), they can also be combined to elucidate finer scale movements (Hanson et al., 2022). Integration of genetic/genomic and chemical markers will continue to benefit from analytical and technological advances within each field (see *Applications: Now and in the future*: *Future directions and conclusions*), and more sophisticated modeling frameworks, enabling high‐resolution insights into connectivity, adaptive variation, and environmentally or genetically driven movement patterns.

### Genetic approaches combined with tagging and telemetry

Studies combining genetic methods with applied tags, generally extract complementary information on connectivity using method‐specific analytical approaches, with genetics often used to infer an individual's origin, which is often unknown when the organism is tagged (Matley et al., 2023). By coupling these techniques, it is possible to link movement behavior (e.g., migration routes, duration, timing) to genetic differentiation, shedding light onto the processes underpinning population structure and dynamics (Michalsen et al., 2014; Minett et al., 2021; Östergren et al., 2012; Sherman et al., 2020). It also increases our understanding of evolutionary biology, offering insights into genotypic and phenotypic diversity, and hence population‐ and ecosystem‐level traits, genomic and behavioral barriers within populations (Barth et al., 2019), and phenomena such as fisheries‐induced evolution (Ward et al., 2016). Looking forward, advances in genomic sequencing, eDNA applications, and real‐time tagging and telemetry will allow further exploration of species' interactions and functional genomics, expanding our understanding of how genetic variation and movement behaviors shape ecological processes, connectivity and adaptive responses to environmental change (Müller et al., 2023).

### Chemical markers combined with tagging and telemetry

Given the temporal constraints of electronic tagging due to battery life and memory capacity, combining their detailed movement patterns (typically for <2 years during adult life) with lifetime estimates of habitat use derived from chemical markers provides valuable insights into MFC across life stages (Baker et al., 2019). Combining tagging and chemical approaches can also provide insights into interindividual variability in resource use, and thus inform conservation (Daban et al., 2024) and monitoring programs (Vander Zanden et al., 2015).

Although difficult to implement, studies that combine chemical records with electronic tag observations from the same individual animal are crucial for validating methods and understanding their limitations (Darnaude & Hunter, 2018; Hüssy et al., 2024; Le Luherne et al., 2022). Improved calibration of chemical tracer data is particularly important because chemical signals often reflect the combined effects of multiple environmental and biological processes rather than a single driver (Doubleday et al., 2025; Sturrock et al., 2012). Advances will also come from expanding chemical analyses to nonlethally sampled tissues, so that tagged individuals do not need to be recaptured in order to obtain a tissue sample, and/or to allow repeat sampling of a tagged individual through time (e.g., for bulk tissue isotopes) for closer temporal alignment with associated tagging data. Finally, stronger coordination among researchers working on MFC would help to identify and share complementary datasets from long‐term tagged individuals, maximizing the value of existing samples and accelerating integrative research.

### Numerical modeling combined with chemical markers

The spatial and temporal resolution of MFC estimates derived from chemical markers can be increased by coupling this method with biophysical models to understand connectivity of passively dispersing life stages (Sakamoto et al., 2019), or with individual‐based movement models to better predict tracks of actively migrating animals (Carpenter‐Kling et al., 2019; Derville et al., 2023; Trueman et al., 2019). Different approaches can be used to inform inference from one another, for example, with some otolith chemistry studies designed based on dispersal pathways predicted using biophysical models (Delerue‐Ricard et al., 2019; Gibb et al., 2017; Wright et al., 2018), or otolith‐derived connectivity estimates validated using the outputs from biophysical dispersal models (Berenshtein et al., 2018; Legrand et al., 2019; Rogers et al., 2021). Future advances may rely on more iterative integration of numerical models and chemical tracer data, enabling patterns in chemical markers to be more tightly linked to the environmental processes that underpin them, and improving our ability to generate simulated movements and estimate their associated uncertainty.

### Numerical modeling combined with tagging and telemetry

Few studies have combined numerical modeling and tagging, as these methods are generally used for early versus adult life stages, respectively. For organisms with complex life histories (e.g., fish that use different habitats throughout their life), combining these methods in a sequential manner can provide information on different connections between habitats and life stages, with biophysical modeling informing the link between spawning area and nursery grounds and tagging providing information on the movements of adults (Abecasis et al., 2024). Applications at the community‐level can also inform on the degree of connectivity and modularity allowing the assessment of ecosystem resilience (Paris et al., 2020). Tagged adults can also provide important information on exact spawning locations, which could then be used as release areas in dispersal modeling scenarios. The continued miniaturization of tags in the future will enable tracking earlier life stages and may at some point even allow coupling between observed movement patterns and modeled dispersal trajectories (Lennox et al., 2025).

## APPLICATIONS: NOW AND IN THE FUTURE

Here, we explore case studies where the above‐reviewed methods have been applied to solve real‐life conservation and management challenges, with a particular focus on (1) MPA design, (2) global change predictions relating to climate change and bioinvasions, and (3) fisheries management.

### Incorporating MFC into MPA design

Given its ecological importance, MFC is a critical parameter in the decision processes that support marine spatial management and MPA network design (Beger et al., 2022). Yet, a clear articulation of this need or implementation approach is often still not explicit in most governance rules and recommendations (e.g., European Parliament and Council, 2008; Balbar & Metaxas, 2019). MPA network design often tries to balance “ecologically beneficial” processes such as larval dispersal and adult movements that support recovery from disturbances against the risk of connected reserves being affected by “ecologically detrimental” processes, such as spatially heterogeneous catastrophic events (e.g., oil spills, disease outbreaks) (Almany et al., 2009; Pérez‐Ruzafa et al., 2017). Similarly, the spatial scales at which connectivity between MPAs manifest are highly variable, depending on the dispersal mechanisms involved, ranging from a few kilometers for most adult movements (Harmelin‐Vivien et al., 2008; Pérez‐Ruzafa et al., 2008), typically tens to hundreds of kilometers for larval dispersal (D'Aloia et al., 2015; Palumbi, 2004), to hundreds to thousands of kilometers for evolutionarily relevant gene flow (Manel et al., 2019).

Capturing the multitude of conservation targets, species, and MFC spatial scales in the planning of MPA networks is complex (Beger et al., 2022) and planners often apply “rules‐of‐thumb” to integrate MFC into decision making, typically in the absence of data (Almany et al., 2009). For example, spatiotemporal heterogeneity can support biodiversity at the genetic and taxonomic level (Pérez‐Ruzafa et al., 2019), relying on connectivity processes to facilitate genetic exchange whilst maintaining the complexity needed to safeguard against broad‐scale impacts. Changes in connectivity caused by climate change, fishing, or other anthropogenic activities can significantly influence flows into and out of MPAs, and the ability of networks to support resilient metapopulations. As such, marine spatial planning is increasingly considering future effects of climate change (Álvarez‐Romero et al., 2018; Andrello et al., 2015).

Several approaches can be used to incorporate MFC data into marine spatial planning. Typically, MFC data are incorporated into decision support tools as mean movements across years, aiming to capture the general direction and strength of flow among areas (Álvarez‐Romero et al., 2018; Beger et al., 2015; Daigle et al., 2020; Magris et al., 2018). Connectivity data can also be incorporated as spatial dependencies (Beger et al., 2015) or with graph‐theoretic features (Magris et al., 2018), yielding broadly similar planning outcomes when larger areas can be protected (Muenzel et al., 2023). In practice, the difficulties obtaining, analyzing, and interpreting MFC data from any method means that MFC is rarely considered as part of MPA design (Balbar & Metaxas, 2019; Beger et al., 2022). When it is, MFC is most commonly represented by larval tracking models (Álvarez‐Romero et al., 2018; Beger et al., 2015; Daigle et al., 2020; Magris et al., 2018), sometimes performed in tandem with SDMs (Combes et al., 2021; Kenchington et al., 2019). However, for MPA network design under the California Marine Life Protection Act, spatial population models of multiple species were used, capturing both adult movement and larval dispersal (Botsford et al., 2014; Kaplan et al., 2019), and there are growing numbers of examples using biotelemetry (Beger et al., 2015; Lea et al., 2016) and genetics (Beger et al., 2014). Genetic methods are more often used in the context of establishing lack of connectivity, or genetic breaks, and are associated with the delineation of management units or isolated populations to represent in management (von der Heyden et al., 2014) and spatial planning (Beger et al., 2014; Nielsen et al., 2020). While chemical markers can be used to determine critical habitats and larval sources (Di Franco et al., 2012; Swearer et al., 1999), they are still rarely used directly in spatial planning. However, Almany et al. (2007) successfully used transgenerational chemical marking of otoliths to assess the appropriateness of an existing MPA, showing that local recruitment of reef fish larvae was more common than previously thought.

The integration of genetic methods with biophysical modeling is showing particular promise for informing marine spatial management (Jahnke & Jonsson, 2022). For example, Jahnke et al. (2020) combined biophysical modeling and genetic analyses to predict connectivity patterns and extinction risk of eelgrass in Swedish fjords, recommending optimal spatial management units aligned with genetic structure, and identifying meadows that maximize the positive impact of restoration on metapopulation connectivity. Similarly, Pujolar et al. (2013) combined genetic methods and biophysical modeling of white seabream to evaluate the effectiveness of an established MPA. Both methods suggested high connectivity rates between neighboring protected and non‐protected areas, highlighting the potential benefits of “spillover effects” as well as potential links to other MPAs at a regional scale. Finally, integration of telemetry data and tissue stable isotopes was used to estimate the habitat use of undulate skates and to propose a new MPA zonation plan (Daban et al., 2024).

### Incorporating MFC into global change predictions

Being able to reliably predict how ecosystems will respond to rapid global change is an important first step towards designing effective management actions that maintain or restore key ecosystem services. Here, we focus on case studies that have used the methods outlined in this paper to understand and predict the influence of climate change on MFC patterns, and to predict and manage marine bioinvasions (Giakoumi et al., 2019).

Generally, discussions around the impacts of climate change on MFC focus on how warming might affect organism survival and connectivity rates. For example, rising water temperatures are generally predicted to reduce the development time and survival of pelagic larval stages (O'Connor et al., 2007; Young et al., 2018), reducing dispersal distances and connectivity rates. Habitat deterioration can also decrease MFC (Gerber et al., 2014), and increased acidification could reduce the growth and survival of calcifiers such as corals and coccolithophores (Leung et al., 2022). However, climate change and anthropogenic activities can also increase MFC and bioinvasion rates, for example, by creating new thermally suitable “corridors” and changing circulation patterns (Booth et al., 2007; Coleman et al., 2017; Poloczanska et al., 2016), or by direct transport of organisms (vector‐driven connectivity) via flotsam and shipping (Minton et al., 2015; Rech et al., 2016). Of course, such increases in potential connectivity are conditioned by biological factors controlling the effective recruitment and survival in the recipient habitat (Booth et al., 2007; Félix‐Hackradt et al., 2018; Seebens et al., 2013). By understanding the mechanisms underpinning past bioinvasions, we can better predict future spread and potential impacts; however, there is still relatively little work in this direction (Lo Brutto et al., 2011).

Biophysical models are most frequently used to predict the impacts of climate change on MFC (Coleman et al., 2017; Vogt‐Vincent et al., 2023) and show great potential to become even more powerful when combined with other models and accounting for physiological constraints and biological interactions (Kotta et al., 2019; Young et al., 2018). Tissue chemical markers can also be used to establish thermal performance curves and combined with ocean model projections to predict when species are likely to be excluded from current strongholds under differing temperature and emissions scenarios (Trueman et al., 2023). Predicted changes in connectivity and species distribution are already being applied widely to adapt fisheries management to a changing climate (Pinsky & Mantua, 2015). There is also growing interest in using climate and MFC predictions to design assisted gene flow (sensu Aitken & Whitlock, 2013), involving the introduction of climate‐resilient genotypes to promote population persistence beyond what might be expected to occur under natural connectivity levels (e.g., Quigley et al., 2019).

For understanding bioinvasion dynamics, SDMs are often used to ascertain the environmental factors explaining past versus present geographic ranges (Carlos‐Júnior et al., 2015). Such models can be useful for invasive species management by identifying current and future hotspots of invasion risk under alternative climate change scenarios (Lyons et al., 2020).

Global change predictions are increasing using integrated MFC methods—mostly genetics (Geller et al., 2010; Rius et al., 2015), SDMs (Battini et al., 2019), and oceanographic modeling—to predict future changes in MFC and biogeography (van Rees et al., 2022). This method combination has been used to understand the spread of an invasive comb jelly, finding that ocean currents can facilitate the reseeding of genotypes over large distances following localized extinctions (Jaspers et al., 2018). Similarly, Richardson et al. (2016) used population genetics, plankton sampling, eDNA, and hydrodynamic modeling to study the spread of a sea star in southeast Australia, finding that both anthropogenic and natural dispersal vectors contributed to its range expansion. Genetic markers (SNPs) and acoustic tagging have also been combined to ascertain dispersal patterns of invasive trout in the Falkland Islands, suggesting a single source population, with its spread associated with widespread adult movements (Minett et al., 2021).

### Incorporating MFC into fisheries management

Many fish species are characterized by high dispersal potential during egg and larval stages, and high levels of mobility as adults, so their distribution can change dynamically (Bruneel et al., 2018). This creates obvious challenges with defining stock boundaries and is a critical issue in fisheries' management (Beverton & Holt, 1957; Cadrin & Secor, 2009; Goethel & Berger, 2017). Furthermore, climate change (see above) can further complicate matters by driving range shifts and altering connectivity rates (Pinsky & Mantua, 2015). Models commonly used in fisheries management (e.g., catch quota, size limits, protection measures) typically simplify the stock structure, resulting in mismatches between management units and biological populations (Kerr et al., 2017). In reality, fish populations are often structured as metapopulations, comprising a series of populations connected through the exchange of individuals (Hidalgo et al., 2019). When catch limits are set without regard for this structure and connectivity patterns, the risk of overharvesting smaller subunits increases, and system productivity is more likely to be incorrectly estimated (Goethel & Berger, 2017). In recent decades, the growing interest in MFC has prompted managers to develop guidelines with best practices that account for spatial dynamics (Cadrin et al., 2023) and to gradually incorporate these processes into fisheries assessment frameworks (Hidalgo et al., 2019).

Multiple methods have been used in stock identification (i.e., the natural “breakpoints” in connectivity) to support fisheries management (Cadrin et al., 2014). Traditionally, these relied on approaches such as morphometrics (Beaumont & Croucher, 2006), otolith shape (Smoliński et al., 2020), and parasite composition (MacKenzie & Abaunza, 1998), while contemporary approaches focus more on genetics, otolith chemistry, tagging, and modeling, as discussed below.

The use of genetics in fisheries management primarily focuses on population structure and the analysis of mixed‐stock fisheries (Bekkevold et al., 2023). For example, a single gene locus has transformed the management of two cod stocks by allowing managers to rapidly assess their proportions in mixed catches (Dahle et al., 2018). High‐throughput sequencing technologies have been instrumental in the use of genetics in fisheries management, increasing the availability of suitable markers and the cost‐effectiveness of the approach, in turn allowing for greater sampling coverage (Farrell et al., 2022). In the near future, genetic tools will likely be used to assign catches to different population groupings in “real‐time” (Andersson et al., 2024). Chemical markers can also reveal stock structure and directly inform fisheries management actions (Reis‐Santos et al., 2023; Tanner et al., 2016). For example, otolith elemental concentrations were used to assess connectivity patterns of cod (Campana et al., 1999) and *Sebastes* spp. (Campana et al., 2007), resulting in fisheries closures and updated management boundaries, respectively. Otolith core chemistry is also routinely used to estimate mixing rates between West Atlantic and Mediterranean bluefin tuna stocks (Kerr et al., 2010; Rooker et al., 2014). Numerical modeling can also inform fisheries management, for example by improving recruitment predictions and the relative importance of ocean currents versus species' biology (self‐recruitment) (Hidalgo et al., 2019). Finally, tagging studies provided early evidence of fish population structure, supporting important concepts of life cycle closure and the unit stock (Harden Jones, 1968). Today, large‐scale mark–recapture programs continue to provide data to support fisheries management (Ono et al., 2022), and increasingly also electronic tagging programs (Crossin et al., 2017; Lowerre‐Barbieri et al., 2019).

In fisheries management, method integration is common, as it is widely acknowledged that stock delineation is most accurate when synthesizing information from multiple techniques (Begg & Waldman, 1999; Cadrin et al., 2023). An interdisciplinary approach maximizes the power to detect spatial structure when it exists (Abaunza et al., 2008; Reis‐Santos et al., 2018; Sarakinis et al., 2024) and to provide greater certainty that a stock is truly a homogeneous unit if structure is not detected (Papetti et al., 2013). A classic success story is the integrated assessment model of Atlantic bluefin tuna that combines otolith chemistry, conventional tagging, catch data, and satellite tag estimates of movement across management units (Taylor et al., 2011). Divergent views of stock structure among methods can help to determine the spatiotemporal scales at which differentiation occurs (Cavole et al., 2023; Taillebois et al., 2017), but they can also complicate interpretation, making it difficult to translate into management. To address this, Welch et al. (2015) developed an Integrated Stock Definition (ISD) framework to synthesize complex information and facilitate clear communication with management stakeholders. The ISD framework can include a semiquantitative stock structure index, valuable for estimating stock boundaries on a weight‐of‐evidence basis (Izzo et al., 2017) but does not (yet) support quantitative stock discrimination or stock composition analysis (Cadrin et al., 2023).

In the future, full method integration (*Method integration*: *Genetic approaches combined with numerical modeling*), combining disparate datasets within a single model, for example using machine learning (Brophy et al., 2020; Chen et al., 2018) or Bayesian approaches (Smith & Campana, 2010), will greatly support stock identification (Cadrin et al., 2023). It will also help to forecast future stock dynamics under alternate climate and ocean circulation scenarios, providing a basis for evolutionary‐based fisheries management (e.g., biophysical modeling and genomics; Baltazar‐Soares et al., 2018; and chemical markers and genetics; Snoeijs‐Leijonmalm et al., 2024).

### Future directions and conclusions

The future of MFC research lies in embracing interdisciplinary approaches and developing comprehensive models that effectively integrate diverse data types at multiple spatiotemporal scales (Darnaude et al., 2024). Such a methodological toolkit will allow scientists to address increasingly complex questions around ecosystem connectivity and metacommunity dynamics, and predict the outcomes of alternative management and conservation strategies under differing climate change scenarios (Gardner et al., 2024; Goethel & Cadrin, 2021; Zipkin et al., 2021).

To improve the accuracy and useability of MFC assessments, we recommend a two‐step approach. First, we advocate for the continued advancement of methods and better quantification of their uncertainties and limitations. Promising developments in the field of tagging include ongoing work in tag miniaturization and biotelemetry receiver coverage, for example through the proliferation of telemetry networks (e.g., OTN). This could be furthered in the future by repurposing infrastructural networks such as cabled observatories, neutrino telescopes and offshore decommissioning structures (Aguzzi et al., 2019). Innovations in the field of genetics include the rapid development of high‐throughput whole genome sequencing at decreasing costs (Andrews et al., 2016). This, combined with increasing computational capacities and coverage of genetic reference libraries, is supporting rapid advances in model‐based demographic reconstructions and seascape genomics (Riginos et al., 2016). For chemical markers, technological advancements improving instrument detection limits and tissue sampling methods are allowing analysis of ever smaller samples (i.e., increasing temporal resolution), and studies are increasingly integrating markers and tissue types within individuals to gain a more complete picture of animal movement and its drivers (Bell‐Tilcock et al., 2021; Reis‐Santos et al., 2023; Walther & Torrance, 2024). The continued development and improvement of global isoscapes and chemoscapes are also supporting growth in MFC and food traceability research (Doubleday et al., 2022; Trueman & St John Glew, 2019); however, there is an ongoing need for validation studies to confirm the mechanisms underpinning tissue signatures (Reis‐Santos et al., 2023). Finally, future innovations in numerical modeling include increasing computing power to improve the speed and accuracy of oceanographic models, particularly around coastal and estuarine areas, and increased training of ecologists in computer simulation skills to foster new collaborations with physical oceanographers. While forecast modeling is frequently called upon to predict future scenarios, we also encourage the scientific community to not neglect the past. The combination of numerical modeling with chemical and genetic analysis of museum and archaeological specimens remains an underutilized opportunity to understand the impacts of environmental change on marine ecosystems over historical and geological timescales (Agiadi et al., 2024; Disspain et al., 2016).

Second, we urge the development of powerful, user‐friendly tools to integrate methods, while appropriately propagating error and weighting different data sources (e.g., Gaggiotti, 2017; Moritz et al., 2009; Vanhatalo et al., 2020). These algorithms would ideally evaluate the most effective methods to meet specific objectives, while also appropriately handling missing information and uncertainty (Hector et al., 2024; Martin et al., 2024). MFC modeling is increasingly moving beyond single species' assessments, requiring increasingly complex models to upscale connectivity estimates to the community level (Moritz et al., 2009). Thus, a major breakthrough will involve harnessing AI to reliably and systematically aggregate and analyze MFC information across taxa, systems, and methods, particularly extracting spatial data in a robust but user‐friendly way (Polak & Morgan, 2024). Exciting new developments in this sphere include Digital Twin of Ocean (DTO) strategies that utilize multiple classes of data and could be used to simulate changes in MFC patterns under future climate scenarios. However, an essential first step to method integration and broader uptake of connectivity in marine management and conservation is increasing the understanding of (and capacity to implement) the various methods highlighted in this review—both by scientists and practitioners.

In summary, we hope that this methodological roadmap helps to explain the main methods used to estimate MFC patterns and to support researchers trying to move between often‐siloed research fields. The future advancement and integration of MFC research will rely on knowledge exchange, capacity building, and data sharing, supported by open‐access databases (e.g., https://mico.eco/, www.movebank.org, https://isobank.tacc.utexas.edu/) and global networks (e.g., OTN, SEA‐UNICORN), supported by simple, transparent and user‐friendly tools, and effective stakeholder engagement to maximize uptake and impact. Diverse collaborations will also help us transition from the single species focus still so often used today towards community‐ and ecosystem‐based approaches, and to build a better understanding of the mechanisms driving MFC and its consequences for ecosystem function. Strengthening cross‐scale governance structures and social‐ecological linkages will also be important to incorporate improved MFC knowledge into management and conservation, with multiple examples already available for nearshore environments and estuaries (Keeley et al., 2022; Sayles & Baggio, 2017). Ultimately, embracing more holistic, integrated approaches will improve the accuracy and widespread application of connectivity data, increasing its value and potential to achieve far broader goals in marine spatial planning and conservation.

## CONFLICT OF INTEREST STATEMENT

The authors declare no conflicts of interest.

## Supporting information


Appendix S1.

## Data Availability

Data and a detailed methods table (Sturrock & Tanner, 2026b) are available in Figshare at https://doi.org/10.6084/m9.figshare.31026817. A complete list of the literature used for the review, including DOIs and unique identifiers, is available in Figshare in Sturrock and Tanner (2026a) at https://doi.org/10.6084/m9.figshare.31325419. Details on the methods and searches used to select the literature included in this paper are provided in Appendix S1: Section S2.
