# Supplementary material for: Methods to estimate marine functional connectivity: A primer
Source: Ecol Appl. 2026 Jun 24;36(4):e70273. doi: 10.1002/eap.70273 (PMC13291831; doi:10.1002/eap.70273)
Supplement: Supplementary file 1 — Appendix S1. [file EAP-36-e70273-s001.pdf]

Appendix S1

Methods to estimate marine functional connectivity: A primer

Anna M. Sturrock, Susanne E. Tanner, Sophie Arnaud-Haond, Jacapo Aguzzi, Francisco R. Barboza, Maria Beger, Andreu Blanco, Deirdre Brophy, Marta Carreton, Amber-Robyn Childs, Federica Costantini, Oscar E. Gaggiotti, Bronwyn M. Gillanders, José M. González-Irusta, Katell Guizien, Tamar Guy-Haim, Stefanie Haase, Ewan Hunter, Jonne Kotta, Geneviève Lacroix, Rafet Ç. Öztürk, Angel Pérez-Ruzafa, Patrick Reis-Santos, Cynthia Riginos, Gil Rilov, Buki Rinkevich, Guiomar Rotllant, David H. Secor, Szymon Smoliński, Clive N. Trueman, Benjamin D. Walther, Audrey M. Darnaude

Ecological Applications

Section S1 – Ancillary methods to support MFC research

Here, we provide an overview of methods that support MFC estimates but do not provide quantitative movement or connectivity estimates.

Presence/absence: direct observation

Presence/absence data provides valuable information for parameterizing and validating numerical models (*An overview of connectivity estimation methods: Numerical modeling* section in main text), but can also be used in isolation to produce a snapshot of species distributions by interpolating presence/absence observations using distance-based kriging techniques (Cressie, 1993). These snapshots can be used to infer connectivity patterns when repeated seasonally or annually over the same area. Presence/absence data can be generated by direct sampling of organisms (e.g., trawling, visual surveys, bioacoustic records, cores). Technological advancements in video and time-lapse cameras have further improved our ability to track individuals or groups of individuals to understand patterns in connectivity and collective movement behaviour (Aguzzi et al., 2019; Rountree et al., 2020). For example, networks of cabled camera observatories that synchronise image acquisition (Aguzzi et al., 2020) can provide counts through time and space, used to evidence onset of active dispersal of fish shoals in benthic (Aguzzi et al., 2011) and pelagic (Chatzievangelou et al., 2021) realms.

Presence/absence: genetic approaches

Presence/absence is now also increasingly inferred via environmental DNA (eDNA), eRNA and DNA metabarcoding (Bucklin et al., 2016; Pawlowski et al., 2018; Weigand et al., 2019). eDNA represents short DNA fragments collected from water and sediment samples that include

the genetic material shed from living and non-living organisms, while DNA metabarcoding analyses complex biological samples containing all organisms in the community (e.g. plankton). These three methods used include Sanger sequencing, quantitative or Real Time PCR (qPCR/RT-PCR), Next-Generation Sequencing (NGS) and Environmental Shotgun Sequencing (ESS), with the resulting sequences compared to reference databases such as NCBI GenBank, BOLD, Silva, PR2 and Metazoogene.

eDNA, eRNA and DNA metabarcoding provide a fast and accurate method for assessing the presence of taxa in different sampling locations to support MFC estimation, as well as more broadly to estimate biodiversity (α and β) and intraspecific diversity, including taxa that are difficult to identify using morphological methods (Ruppert et al., 2019; Thomsen & Willerslev, 2015), such as small, rare or cryptic species (Guy-Haim et al., 2022) and early life stages (Carreton et al., 2019; Couton et al., 2019; Ershova et al., 2019; Govender et al., 2022). The study of phylogeographic features for hundreds of species simultaneously, termed metaphylogeography, can provide valuable information on biogeography, connectivity, and dispersal patterns (Adams et al., 2019; Thomasdotter et al., 2023; Turon et al., 2020). While eDNA and DNA metabarcoding hold great promise for improving spatial and temporal biological monitoring in aquatic ecosystems (Mirimin et al., 2021), the comprehensive understanding required for practical application in real-world settings is generally still lacking (Harrison et al., 2019; Kirtane et al., 2021; Sassoubre et al., 2016). For example, sequencing errors (Edgar & Flyvbjerg, 2015; Turon et al., 2020) can severely bias intraspecies patterns by artificially inflating haplotype diversity, while sequence databases and reference libraries often lack key marine taxa and rigorous quality control (Paz & Rinkevich, 2021). Also, a better understanding of eDNA degradation rates and transport dynamics in aquatic environments is essential to better interpret detections (or lack of) and their spatial configuration, with the

geographic resolution often limited to tens to hundreds of kilometres (Harrison et al., 2019). While DNA metabarcoding circumvents some of these limitations by analysing the entire community (Beng & Corlett, 2020) the resulting data is semiquantitative, and extrapolation to abundances is nontrivial. Finally, these techniques do not per se give information about life stage and sex, which might be desirable in MFC modeling.

Morphometrics and other natural markers

In the absence of connectivity, groups of animals can develop differences in morphology or ecological traits. These differences can be driven by genetic differences caused by random drift or adaptation to location conditions, plasticity and/or natural tags or "biotags" imparted directly from the environment (e.g. chemical markers, see above, or parasite loads). The resulting differences in tags or traits can be used to describe population structure (i.e. lack of connectivity, as described above in Sections 2.2 and 2.3). Complementary to these approaches are the use of organism morphometrics (Silva, 2003), meristics (Watanabe et al., 2009)) and otolith shape (Neves et al., 2023). In addition, differences in parasite communities and genetic signatures can be used to infer population structure and MFC patterns of the host animal (Catalano et al., 2014; MacKenzie & Abaunza, 1998). As with all these methods, if the population-level differences are well described, then they can be used to infer connectivity and mixed stock composition during brief periods of mixing (Campana et al., 2000). Finally, note also that for marine megafauna, individual animals can be tracked using individually-identifiable external features such as whale flukes, without any need for handling of the animal (Marcos et al., 2022).

At the population level, these natural markers tend to vary over large spatial scales and at coarse resolution. Similar to chemical markers, differences in natural markers among groups suggests

a lack of connectivity, but a lack of difference does not necessarily suggest connectivity nor infer the degree of connectivity between them, as these differences can simply reflect similar environmental conditions in two different places. In addition, many of these methods are labour-intensive and may require lethal sampling (e.g. otolith morphometrics), specific storage conditions (e.g. meristics) or specialised taxonomic expertise (e.g. parasite loads).

Section S2 – Methods used for literature searches

This review was largely derived from ad-hoc literature searches by each coauthor and their expert knowledge on the latest methods and research relevant to this review. The list of references including their unique identifiers is available on Figshare (doi: 10.6084/m9.figshare.31325419). To generate Figure 5, four Web of Science Topic searches (articles only) were conducted on April 4, 2024 using the following terms: "Allozyme", Microsatellite", "mtDNA OR cpDNA" and "SNPs". Total number of articles identified were 7998, 39289, 75587, and 78924, respectively, separated by publication year (2023 and 2024 merged). The total number of articles found between 1970 and 1985 was 690 (89.5% allozymes, 10% mtDNA or cpDNA, 0.5% microsatellites).

References

Adams, C. I. M., Knapp, M., Gemmell, N. J., Jeunen, G.-J., Bunce, M., Lamare, M. D., & Taylor, H. R. (2019). Beyond Biodiversity: Can Environmental DNA (eDNA) Cut It as a Population Genetics Tool? *Genes*, *10*(3), 192.

Aguzzi, J., Chatzievangelou, D., Company, J. B., Thomsen, L., Marini, S., Bonofiglio, F., … Gaughan, P. (2020). The potential of video imagery from worldwide cabled

observatory networks to provide information supporting fish-stock and biodiversity assessment. *ICES Journal of Marine Science*, *77*(7–8), 2396–2410.

Aguzzi, J., Chatzievangelou, D., Marini, S., Fanelli, E., Danovaro, R., Flögel, S., … Company, J. B. (2019). New High-Tech Flexible Networks for the Monitoring of Deep-Sea Ecosystems. *Environmental Science & Technology*, *53*(12), 6616–6631.

Aguzzi, J., Sbragaglia, V., Sarriá, D., García, J. A., Costa, C., Río, J. D., … Sardà, F. (2011). A New Laboratory Radio Frequency Identification (RFID) System for Behavioural Tracking of Marine Organisms. *Sensors*, *11*(10), 9532–9548.

Beng, K. C., & Corlett, R. T. (2020). Applications of environmental DNA (eDNA) in ecology and conservation: Opportunities, challenges and prospects. *Biodiversity and Conservation*, *29*(7), 2089–2121.

Bucklin, A., Lindeque, P. K., Rodriguez-Ezpeleta, N., Albaina, A., & Lehtiniemi, M. (2016). Metabarcoding of marine zooplankton: Prospects, progress and pitfalls. *Journal of Plankton Research*, *38*(3), 393–400.

Campana, S. E., Chouinard, G. A., Hanson, J. M., Fréchet, A., & Brattey, J. (2000). Otolith elemental fingerprints as biological tracers of fish stocks. *Fisheries Research*, *46*(1), 343–357.

Carreton, M., Company, J. B., Planella, L., Heras, S., García-Marín, J.-L., Agulló, M., … Roldán, M. I. (2019). Morphological identification and molecular confirmation of the deep-sea blue and red shrimp *Aristeus antennatus* larvae. *PeerJ*, *7*, e6063.

Catalano, S. R., Whittington, I. D., Donnellan, S. C., & Gillanders, B. M. (2014). Parasites as biological tags to assess host population structure: Guidelines, recent genetic advances and comments on a holistic approach. *International Journal for Parasitology: Parasites and Wildlife*, *3*(2), 220–226.

Chatzievangelou, D., Bahamon, N., Martini, S., del Rio, J., Riccobene, G., Tangherlini, M., … Aguzzi, J. (2021). Integrating Diel Vertical Migrations of Bioluminescent Deep Scattering Layers Into Monitoring Programs. *Frontiers in Marine Science*, *8*, 661809.

Couton, M., Comtet, T., Le Cam, S., Corre, E., & Viard, F. (2019). Metabarcoding on planktonic larval stages: An efficient approach for detecting and investigating life cycle dynamics of benthic aliens. *Management of Biological Invasions*, *10*(4), 657–689.

Cressie, N. (1993). Statistics for Spatial Data. In *Applied Probability and Statistics* (Revised reprint of the 1991 edition). John Wiley & Sons Inc.

Edgar, R. C., & Flyvbjerg, H. (2015). Error filtering, pair assembly and error correction for next-generation sequencing reads. *Bioinformatics*, *31*(21), 3476–3482.

Ershova, E. A., Descoteaux, R., Wangensteen, O. S., Iken, K., Hopcroft, R. R., Smoot, C., … Bluhm, B. A. (2019). Diversity and Distribution of Meroplanktonic Larvae in the Pacific Arctic and Connectivity With Adult Benthic Invertebrate Communities. *Frontiers in Marine Science*, *6*, 490.

Govender, A., Groeneveld, J., Singh, S., & Willows-Munro, S. (2022). Metabarcoding of zooplankton confirms southwards dispersal of decapod crustacean species in the western Indian Ocean. *African Journal of Marine Science*, *44*(3), 279–289.

Guy-Haim, T., Velasquez, X., Terbiyik-Kurt, T., Di Capua, I., Mazzocchi, M., & Morov, A. (2022). A new record of the rapidly spreading calanoid copepod Pseudodiaptomus marinus (Sato, 1913) in the Levantine Sea using multi-marker metabarcoding. *BioInvasions Records*, *11*(4), 964–976.

Harrison, J. B., Sunday, J. M., & Rogers, S. M. (2019). Predicting the fate of eDNA in the environment and implications for studying biodiversity. *Proceedings of the Royal Society B: Biological Sciences*, *286*(1915), 20191409.

Kirtane, A., Wieczorek, D., Noji, T., Baskin, L., Ober, C., Plosica, R., … Sassoubre, L. (2021). Quantification of Environmental DNA (eDNA) shedding and decay rates for three commercially harvested fish species and comparison between eDNA detection and trawl catches. *Environmental DNA*, *3*(6), 1142–1155.

MacKenzie, K., & Abaunza, P. (1998). Parasites as biological tags for stock discrimination of marine fish: A guide to procedures and methods. *Fisheries Research*, *38*(1), 45–56.

Marcos, D., Kierdorf, J., Cheeseman, T., Tuia, D., & Roscher, R. (2022). A Whale's Tail—Finding the Right Whale in an Uncertain World. In A. Holzinger, R. Goebel, R. Fong, T. Moon, K.-R. Müller, & W. Samek (Eds.), *xxAI - Beyond Explainable AI* (Vol. 13200, pp. 297–313). Springer International Publishing.

Mirimin, L., Desmet, S., Romero, D. L., Fernandez, S. F., Miller, D. L., Mynott, S., … Aguzzi, J. (2021). Don't catch me if you can – Using cabled observatories as multidisciplinary platforms for marine fish community monitoring: An in situ case study combining Underwater Video and environmental DNA data. *Science of The Total Environment*, *773*, 145351.

Neves, J., Veríssimo, A., Múrias Santos, A., & Garrido, S. (2023). Comparing otolith shape descriptors for population structure inferences in a small pelagic fish, the European sardine Sardina pilchardus (Walbaum, 1792). *Journal of Fish Biology*, *102*(5), 1219–1236.

Pawlowski, J., Kelly-Quinn, M., Altermatt, F., Apothéloz-Perret-Gentil, L., Beja, P., Boggero, A., … Kahlert, M. (2018). The future of biotic indices in the ecogenomic era: Integrating (e)DNA metabarcoding in biological assessment of aquatic ecosystems. *Science of The Total Environment*, *637–638*, 1295–1310.

Paz, G., & Rinkevich, B. (2021). Gap analysis of DNA barcoding in ERMS reference libraries for ascidians and cnidarians. *Environmental Sciences Europe*, *33*(1), 4.

Rountree, R. A., Aguzzi, J., Marini, S., Fanelli, E., De Leo, F. C., Del Rio, J., & Juanes, F. (2020). Towards an optimal design for ecosystem-level ocean observatories. In S. J. Hawkins, A. L. Allcock, A. E. Bates, A. J. Evans, L. B. Firth, C. D. McQuaid, … P. A. Todd (Eds.), *Oceanography and Marine Biology* (1st ed., pp. 79–105). CRC Press.

Ruppert, K. M., Kline, R. J., & Rahman, M. S. (2019). Past, present, and future perspectives of environmental DNA (eDNA) metabarcoding: A systematic review in methods, monitoring, and applications of global eDNA. *Global Ecology and Conservation*, *17*, e00547.

Sassoubre, L. M., Yamahara, K. M., Gardner, L. D., Block, B. A., & Boehm, A. B. (2016). Quantification of Environmental DNA (eDNA) Shedding and Decay Rates for Three Marine Fish. *Environmental Science & Technology*, *50*(19), 10456–10464.

Silva, A. (2003). Morphometric variation among sardine (Sardina pilchardus) populations from the northeastern Atlantic and the western Mediterranean. *ICES Journal of Marine Science*, *60*(6), 1352–1360.

Thomasdotter, A., Shum, P., Mugnai, F., Vingiani, M., Dubut, V., Marschal, F., … Costantini, F. (2023). Spineless and overlooked: DNA metabarcoding of autonomous reef monitoring structures reveals intra- and interspecific genetic diversity in Mediterranean invertebrates. *Molecular Ecology Resources*, *23*(7), 1689–1705.

Thomsen, P. F., & Willerslev, E. (2015). Environmental DNA – An emerging tool in conservation for monitoring past and present biodiversity. *Biological Conservation*, *183*, 4–18.

Turon, X., Antich, A., Palacín, C., Præbel, K., & Wangensteen, O. S. (2020). From metabarcoding to metaphylogeography: Separating the wheat from the chaff. *Ecological Applications*, *30*(2).

Watanabe, S., Miller, M. J., Aoyama, J., & Tsukamoto, K. (2009). Morphological and meristic evaluation of the population structure of *Anguilla marmorata* across its range. *Journal of Fish Biology*, *74*(9), 2069–2093.

Weigand, H., Beermann, A. J., Čiampor, F., Costa, F. O., Csabai, Z., Duarte, S., … Ekrem, T. (2019). DNA barcode reference libraries for the monitoring of aquatic biota in Europe: Gap-analysis and recommendations for future work. *Science of The Total Environment*, *678*, 499–524.
